# Select Polyphenol-Rich Berry Consumption to Defer or Deter Diabetes and Diabetes-Related Complications

**DOI:** 10.3390/nu12092538

**Published:** 2020-08-21

**Authors:** Ahsan Hameed, Mauro Galli, Edyta Adamska-Patruno, Adam Krętowski, Michal Ciborowski

**Affiliations:** 1 Clinical Research Center, Medical University of Bialystok, 15-089 Bialystok, Poland; ahsan.hameed@umb.edu.pl (A.H.); edyta.adamska@umb.edu.pl (E.A.-P.); adamkretowski@wp.pl (A.K.); 2 Department of Medical Biology, Medical University of Bialystok, 15-222 Bialystok, Poland; mauro.galli@umb.edu.pl; 3 Department of Endocrinology, Diabetology, and Internal Medicine, Medical University of Bialystok, 15-089 Bialystok, Poland

**Keywords:** berries, metabolic syndrome, precision nutrition, hyperglycemia, hyperlipidemia, diabetes, omics, metabolomics, genomics

## Abstract

Berries are considered “promising functional fruits” due to their distinct and ubiquitous therapeutic contents of anthocyanins, proanthocyanidins, phenolic acids, flavonoids, flavanols, alkaloids, polysaccharides, hydroxycinnamic, ellagic acid derivatives, and organic acids. These polyphenols are part of berries and the human diet, and evidence suggests that their intake is associated with a reduced risk or the reversal of metabolic pathophysiologies related to diabetes, obesity, oxidative stress, inflammation, and hypertension. This work reviewed and summarized both clinical and non-clinical findings that the consumption of berries, berry extracts, purified compounds, juices, jams, jellies, and other berry byproducts aided in the prevention and or otherwise management of type 2 diabetes mellitus (T2DM) and related complications. The integration of berries and berries-derived byproducts into high-carbohydrate (HCD) and high-fat (HFD) diets, also reversed/reduced the HCD/HFD-induced alterations in glucose metabolism-related pathways, and markers of oxidative stress, inflammation, and lipid oxidation in healthy/obese/diabetic subjects. The berry polyphenols also modulate the intestinal microflora ecology by opposing the diabetic and obesity rendered symbolic reduction of Bacteroidetes/Firmicutes ratio, intestinal mucosal barrier dysfunction-restoring bacteria, short-chain fatty acids, and organic acid producing microflora. All studies proposed a number of potential mechanisms of action of respective berry bioactive compounds, although further mechanistic and molecular studies are warranted. The metabolic profiling of each berry is also included to provide up-to-date information regarding the potential anti-oxidative/antidiabetic constituents of each berry.

## 1. Introduction

Diabetes mellitus (DM) is a multifactorial disease with high mortality worldwide. Chronic DM is the eighth-leading cause of deaths globally, responsible for 1.5 million deaths each year [1]. According to the World Health Organization (WHO), in 2013, 381 million adults were diagnosed with DM, which increased to 422 million in 2016 and is expected to double by 2030. Type 1 (T1DM) represents 15% of cases, and the remaining cases are type 2 (T2DM) [1]. T2DM is primarily treated with pharmacotherapeutic drugs, evidence-based alternative approaches, and functional food-based approaches [2]. Pharmacotherapeutic approaches generally consist of monotherapy or binary/poly-therapy, depending on severity. Most physicians use the binary approach and prescribe insulin-secretogenic sulfonylurea drugs and the insulin sensitivity enhancer metformin. Additional drugs address diabetes-induced vascular complications, with the average number of prescribed daily drugs being as high as four [3]. Combined drug therapy is associated with long-term side-effects and other costs, resulting in non-adherence [4]. Moreover, evidence-based alternative approaches may have safety and toxicity issues due to which precision nutrition-based approaches have recently been proposed as alternatives to defer or deter T2DM and its complications.

The provision of individualized dietary and nutritional recommendations is referred to as precision nutrition. Polyphenol-rich fruits (including berries) are the primary components of precision nutrition, and consumption of these fruits, like berries, represent a potential “frontline strategy” for combating T2DM in obese or overweight patients. Substantial evidence suggests that T2DM onset can be prevented or managed by berries and/or berries-derived-tailored dietary intake, exercise, and the maintenance of healthy body weights (BWs) [5]. Therefore, targeted berries-nutrition is considered analogous to an individualized medicinal approach, providing effective and safe nutritional interventions for DM prevention and management. Furthermore, the American Diabetes Association and Dietary Guidelines for Americans also strongly recommend diets rich in anthocyanin and polyphenols to protect against and manage DM [6]. Increasing evidence shows that berry consumption also reduces DM risk, including a recent study showing that Finnish men who regularly consumed berries reduced their T2DM risks by up to 35% [7]. Due to the significance of berry consumption and the lack of comprehensive studies examining berry consumption effects specifically on DM, this study aimed to collect and summarize all studies examining the relationship between berry consumption and DM.

DM is a metabolic syndrome with concordance changes in insulin sensitivity and/or availability. This insulin insensitivity and/or deficiency induces derangements in metabolic pathways related to glucose, lipids, and protein metabolism. Berry, or its byproduct, intake not only opposes these derangements by normalizing the metabolic homeostasis of glucose, lipids, and protein metabolism, but also improves insulin sensitivity and secretary indexes. Therefore, all available in vitro and in vivo studies involving whole berries or berry bioproduct consumption and citing the normalization of insulin signaling, secretion, and sensitivity, restoring the altered glucose, lipid, and protein metabolism, and reduction of oxidative stress and inflammatory cytokines were included. In order to determine the hypoglycemic and hypolipidemic potential of berries, studies that added berries to high-fat (HFD) and high-carbohydrate (HCD) diets, defined as diets with >45% fat and >60% carbohydrates, respectively, were also included. In addition to HFD and/or HCD, disruption of intestinal endothelium and homeostasis resulting in epithelial inflammation, increased permeability (i.e., dysbiosis), and alteration in gut microbial taxonomic composition and diversity (increase in Firmicutes:Bacteroidetes ratio, and reduction in intestinal mucosal barrier dysfunction (IMBD) restoring bacterial families, proteolytic and glycolytic microflora, short-chain fatty acids (SCFA), and organic acids (SCOA) producing microflora) are also considered risk factors to obesity and DM. IMBD associated bacterial families protect the epithelial layer of the intestine whereas SCFA and SCOA played important role in the synthesis and production of immunoglobulins and immune-supportive cytokines to protect against dysbiosis and metabolic disorders. In this context, the impact of berry or berry product intake on the attenuation of obesity-associated disorders and dysbiosis was also reviewed. Studies involving the metabolic fingerprinting of berries were also described to represent the possible number of compounds considered responsible for their antioxidative and antidiabetic actions.

Consequently, this review aimed to discuss scientific evidence regarding a positive role of berry consumption on the prevention or delay of diabetes development and reduction or avoidance of diabetes-related complications. Moreover, a detailed composition of different berries is also presented.

## 2. Methods

**Figure 1:**
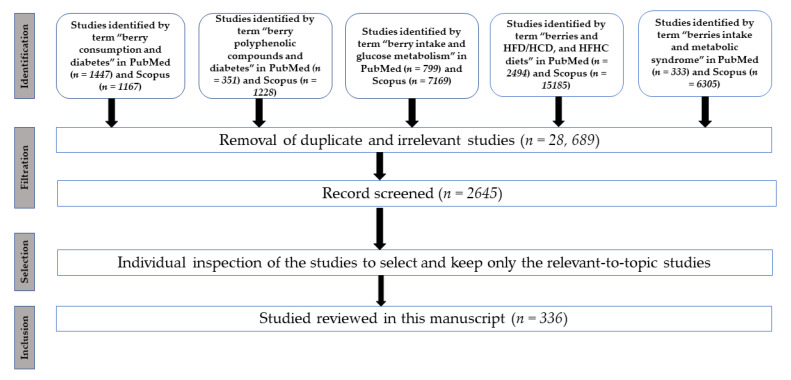
Schematic representation of Preferred Reporting Items for Systematic Reviews and Meta-Analyses (PRISMA) flow diagram collection and selection of studies included in this review. Adapted from Moher, Liberati [8].

Studies examining berry consumption and T2DM were searched for (last time accessed 15 June 2020) in the Medline/PubMed, ScienceDirect/Scopus, and Web of Sciences databases using the following keywords and phrases: berry consumption and diabetes, berry polyphenolic compounds and diabetes, berry intake and glucose metabolism, berries and high-fat diets, berries and high-glycemic diets, metabolic fingerprinting of berries, lipid metabolism and berries, glycemic control, human clinical trials with berries, in vitro/in vivo studies using berries, and individual berry names. The search using these keywords and phrases resulted in more than 3000 articles in said scientific databases, as illustrated in detail in Figure 1. All articles not in line with the objectives of this review article were not considered. Additionally, the articles that were found more than once in these databases were counted once, and after removal of these duplicate and irrelevant records, nearly 2645 publications were thoroughly screened for inclusion eligibility. Finally, 336 publications were found relevant and fit to be reviewed. Only studies examining berries or berry product consumption relative to metabolic syndrome conditions or otherwise DM respective and berry fingerprinting were included. The schematic flow diagram for the selection of studies in this work is presented in Figure 1.

## 3. Blueberries

Blueberries (BlBs) top the list of five fruits recommended by the Food and Agriculture Organization of the United Nations (FAO) against diabetes, cancer, liver disease, anemia, and cardiovascular disease (CVD). Initially, the in vitro antidiabetic activity of BlBs were reported by Barberis et al. [9] and Martineau et al. [10]. Barberis et al. described the reduced amount of glucose absorption in the Caco-2TC7 monolayer human intestinal cell line in the supplementation of phosphate-buffered-saline (PBS) containing BlB juice (BlBJ) prior to glucose stimulation. Martineau et al. [10] used insulin-dependent/independent 3T3-L1, C2C12, and TC-tet cell lines. The overnight incubation of these cells with BlB extracts (BlBEs) enhanced glucose uptake even in the absence of insulin compared to the vehicle-delivered control cell cultures [10]. The basal secretion of insulin from TC-tet cells increased 2.5 times to 7.5 times with increasing glucose amounts from 6 mM to 10 mM. A significant increase in glucose-stimulated insulin secretion (GSIS) was also seen after treating cells with BlBEs [10]. The BlBE adipogenic effects were also examined by assessing lipid formation and accumulation in pre-adipocytes, and BlB treatment was almost as effective as the positive control, rosiglitazone, for lipid accumulation. BlB consumption downregulated the HFD-induced upregulation of total cholesterol (TC), low-density lipoprotein cholesterol (LDL-C), leptin, and inflammatory genes (L-6, TNF-α, inducible nitric oxide synthase (iNOS)), monocyte chemo-attractant protein-1 (MCP-1) (an inflammatory cytokine), peroxisome proliferator-activated receptors γ (PPAR-γ), and fatty acid synthase (FAS) [11,12].

Hypoglycemic and hypolipidemic potential of BlBs or its polyphenol rich products has also been checked in many in vivo studies; Grace et al. [13] fed *streptozotocin* (STZ)-induced diabetic rats diets supplemented with phenolic- and anthocyanin-enriched BlBES. Anthocyanin-enriched diets increased hypoglycemic activity (51%) compared with phenolic-enriched diets (33%) and metformin-treated controls (32%), suggesting that anthocyanins modulated hyperglycemic and hyperlipidemic activities [13]. The supplementation of BlBE increased the beneficial glucose metabolism involved peroxisome proliferator response element (PPRE) (1.3–1.8%), glucose transporter 2 (GLUT-2) (1.5%), and PPAR-γ (1.4%) activities, and reduced the proinflammatory nuclear factor (NF)-κB activity [14]. Furthermore, an increase in the intercellular levels of the mRNA of glucose transporter (GLUT4), insulin receptor substrate-1/2 (IRS-1/IRS-2) (insulin response mediators regarding glucose metabolism), and AMP-activated protein kinase (AMPK) (a key regulator of mitochondrial biogenesis and cellular energy homeostasis) were observed in skeletal muscles, indicating increased glucose uptake [15,16]. BlB metabolites, especially anthocyanins, also promoted glucagon-like peptide-1 (GLP-1) expression and PPAR activity; GLP-1 increases glucose-dependent insulin secretion and pancreatic β-cell proliferation, whereas PPAR and nuclear fatty acid receptors improve IR [17]. Few studies have also shown improved insulin resistance (I) but with conflicting results in reduced BW gains [16,18,19]. However, in human clinical trials, improved insulin sensitivity without BW changes was observed [20]. Thus, insulin sensitivity may increase following BlB consumption, but BlBE may be less effective for modulating weight loss. Besides the BlBE, a few studies have also used the whole BlB fruit to determine its antidiabetic and anti-obesity potential in a group of people at high risk of T2DM (Table 1). BlB smoothie supplementation significantly reduced insulin resistance (IR) [21]. The ad libitum consumption of 100% pure BlBJ showed hypoglycemic activity, suppressing fatty acid synthase (FAS)- and β-oxidation-related gene expression in HFD-fed C57BL/6 mice (Table 1) [12]. Alcohol-free fermented juice, containing 30% BlBJ and 70% blackberry juice, reduced epididymal fat pad weights, percent fat mass, plasma triglyceride, and total cholesterol (TC) levels as well as mean adipocyte diameters and improved fasting blood glucose and GTT levels [22]. In another study, BlBJ consumption increased glucose uptake and inhibited adipogenesis by reducing adiponectin levels in KKKy mice [23]. In addition to BlBJ, BlB powder consumption in sugar-matched/sugar-non-matched smoothies extended the post-prandial glucose response and reduced peak postprandial glucose levels [24]. Diabetes and obesity are inter-linked via chronic inflammatory conditions, where macrophages infiltrate and accumulate in adipose tissue, triggering pro-inflammatory cytokine secretion [25]. BlB supplementation reduced these (pro)-inflammatory cytokine secretions (i.e., NF-κB, interleukin (IL)-10, tumor necrosis factor (TNF)-α, and IL-6 expression) in obese and diabetic mice [25]. BlBE consumption also showed excellent anti-inflammatory effects against soluble vascular cell adhesion molecule-1 (sVCAM-1) (inflammatory biomarker), MCP-1, C-reactive protein (CRP) (acute inflammatory protein), and vascular endothelium [26].

Oxidative stress increases reactive oxygen species (ROS), chemokines, nitric oxides (NOs), adhesion molecules, nuclear factor (IκBα) production, and glycation prior or after diabetes. Human aortic endothelial cells (HAECs) treated with purified BlB anthocyanins (hippuric acid, hydroxyhippuric acid, isovanillic acid-3-sulfate, benzoic acid-4-sulfate, and vanillic acid-4-sulfate) demonstrated reduced ROS, chemokine, NO, adhesion molecule, and IκBα production [27]. In a human clinical trial, post-exercise blueberry BlB consumption decreased manganese superoxide dismutase (Mn-SOD) levels [28]. Li et al. [29] reported anti-oxidative and anti-inflammatory cytokine marker suppression by 19 and 31%, respectively, in adipocytes and macrophages co-cultured with piceatannol, a BlB-derived bioactive compound. Piceatannol also ameliorated malfunctioning insulin-stimulated glucose uptake by upregulating Akt phosphorylation (crucial for IRS activation and hence increasing insulin sensitivity) and forkhead box O1 (FOXO1) (a transcription factor). Pterostilbene, a PPAR-α agonist found in BlB, promoted fatty acid catabolism by upregulating (up to 3%) of AMPK, carnitine palmitoyl transferase-1 (CPT-1) (an enzyme for long-chain fatty acid-LCFA oxidation), acyl-CoA oxidase (ACOX) (enzyme of β-oxidation system), and uncoupling protein-2 (UCP-2) (a protein involved in glucose disposal, insulin secretion, and cellular energy dissipation) expression. AMPK is associated with mitochondrial energy production, and AMPK activation regulates liver cell gluconeogenesis by suppressing glucose-6-phosphatase (G6Pase), phosphoenolpyruvate carboxykinase (PEPCK) (gene involved in glyceroneogenesis and gluconeogenesis), FOXO1, PPAR-γ coactivator 1α (PGC-1α), and glucose production. FOXO1 regulates PEPCK, PGC-1α, and G6Pase expression, thus affecting glucose release [30].

The integration of BlB polyphenols with a HFD also attenuated HFD rendered disorders and dysbiosis. The BlB powder supplementation improved the systematic inflammation and insulin sensitivity by modulating the gut microbial population in rat fed on a HFD [31]. In human, BlB intervention offered the prebiotic-effect by increasing the relative abundance of beneficial fermentative bacterium *Bifdobacterium* spp [32]. The BlB-derived anthocyanins also improved the IMBD restoration by decreasing the population of *E. coli* [33]. More recently, Rodríguez-Daza and Daoust [34] also witnessed that BlB-derived proanthocyanidins did not significantly improve the dysbiosis symbolic Firmicutes:Bacteroidetes ratio, but its supplementation did improve the population of genera (Akkermansia, Adlercreutzia, an unknown genus of order Clostridiales, Peptostreptococcaceae, and Ruminococcaceae) considered responsible for the maintenance and restoration of the colon mucosal barrier. The health promoting role of BlB and its byproducts can be explained further by a comprehensive metabolite profile for BlB/BlBE [10,35,36,37,38,39] and is shown in Table 2. The metabolic fingerprinting reveals BlB/BlBE as a rich source of antioxidative, antidiabetic, anti-inflammatory anthocyanins, proanthocyanidins, epicatechins, aglycons, glycosides, catechins, phenolic acids, chlorogenic acids, caffeic acid derivatives, and quercetin derivatives. Collectively, these studies demonstrated that BlB supplementation protected against HFD/HCD-induced IR hyperglycemia, pro-inflammatory responses, oxidative stress, adipocyte death, and improved insulin sensitivity, with mixed results for HFD-induced BW gain. The identified anthocyanins associated with these activities include glucosides, galactosides, and arabinosides of cyanidin, delphinidin, malvidin, peonidin, and petunidin.

**Table 1:** A comprehensive list of berry interventions and their consequent effects on various levels.

No.	Study Design	Study Subject	Duration	Berry Interventions	Intervention Diet	Significant Findings	Ref.
(I) Blueberries (BlB) (Animal studies)							
1	RCT	C57bl/6J mice (*n* = NS)	12 wk	BlB anthocyanins 300–500 mg/kg.BW.day	LFD (20% kcal from lard fat) and HFD (70%kcal from lard fat)	Lower the blood glucose level and dyslipidemia markers	[13]
2	RCT	Male C57BL/6 mice (*n* = 24)	8 wk	4% (*w/w*) F/D whole BlB powder with HFD provided ad libitum	HFD (60% kcal from fat)	Offer protection against HFD-induced obesity, adipose tissue macrophages inflammatory gene expression, and oxidative stress	[11]
3	RCT	C57/Bl6 mice (*n* = 200)	12 wk	5% or 10% (*w/w*) of whole BlB with HFD provided ad libitum	HFD (45% kcal from fat)	Reduced HFD-induced cellular inflammatory cytokines, chemokines, interleukins, and proinflammatory interferon gamma -producing T-cells	[18]
4	RCT	Male Zucker Fatty and Zucker Lean rats (*n* = 48)	8 wk	4% (*w/w*) F/D whole BlB powder with HFD provided ad libitum	HFD (45% kcal from fat)	Hypolipidemic, Hyper-insulinemic, hypoglycemic and anti-inflammatory	[40]
5	RCT	C57BL/6 mice (*n* = 48)	12 wk	Ad libitum 100% BlBJ with HFD provided ad libitum	LFD (20% kcal from fat) and HFD (45%kcal from fat)	Reduced expressions of inflammatory and FA synthesis genes. Reduced IR and plasma dyslipidemia markers	[12]
6	RCT	C57BL/6 mice (*n* = 72)	8 wk	65.1 ± 1.6 mg cyanidin-3-*O*-glucoside/L (from 30% BlB + 70% blackberry juice available ad libitum)	HFD (60% kcal from fat)	Anti-obesity, hypoglycemic, antidiabetic	[22]
7	RCT	C57BL/6 and KKAy mice (*n* = 20)	4 wk	BlBJ (40–80 mL/kg per day in drinking water)	Normal chow diet	Improved glucose tolerance, reduced glycemic response suggesting increased insulin sensitivity	[23]
8	RCT	Obese Zucker rat (*n* = 20)	8 wk	8% wild BlB diet (WB) provided ad libitum	NA	Downregulated expression and plasma concentrations of NF-kB, TNFα, IL-6, CRP in liver and adipose tissues	[25]
	Human studies						
9	SB and RCT	Obese men and women (*n* = 66)	8 wk	50 g F/DBlB per day	NA	Reduction in plasma oxidized LDL and other plasma lipid oxidation products	[20]
10	DB, PC, RCT	overweight or obese individuals (*n* = 30)	4 wk	4 g of inulin/day from BlB (equivalent to two cups of whole BlB)	NA	Improvement in glycemic response, insulin sensitivity, satiety, serum lipid parameters, and fecal markers of gut microbiota	[41]
11	DB, PC, RCT	Diabetic patients (*n* = 58)	24 wk	160 mg of BlB anthocyanins twice daily	NA	Reduced serum concentration of LDL-C, TG, apolipoprotein, apolipoprotein C-III, lipid and protein oxidation markers with strengthening the inherent antioxidative system	[42]
12	DB, PC, RCT	Healthy adults (*n* = 44)	6 wk	45 g/day F/D BlBpowder	12-oz yogurt and skim milk-based smoothie	Improvement in endothelial function in subjects with metabolic syndrome	[21]
13	DB, CO, RCT,	Healthy human beings (*n* = 17)	4 wk	310–724 mg/kg.BW.day BlBanthocyanin	Sugar-matched smoothie	Extend the postprandial glucose response beyond the period observed for a sugar-matched control	[24]
	(I) Bilberries (BB) (Animal studies)						
1	RCT	Male KK-Ay mice (*n* = 16)	5 wk	27 g of BB extract/kg diet daily	NA	Activation of AMP-activated protein kinase (AMPK) resulting in increased insulin sensitivity, upregulation of glucose transporter GLUT4, suppression of glucose production in liver	[15]
2	RCT	diabetic groups of Wistar rats (*n* = 32)	4 wk	BB extracts 2 g/day by gavage	Normal chow diet	Increased serum insulin, reduced TC, VLDL-C, LDL-C, and TG levels, and prevented HDL-C decline	[17,43]
3	RCT	Brown Norway (BN) rats (*n* = 96)	6 wk	BB extract 100 mg/kg.BW.day	Normal chow diet	Prevent diabetic retinopathy	[44]
4	RCT	Male KM mice (*n* = 60)	5 d	BB extract (containing 42.04% anthocyanins) 200 mg/kg BW·day)	Normal chow diet	Reduced the live damage and oxidative stress markers (ALT, MDA, NO) with improvements in enzymatic antioxidative system (GSH)	[45]
5	RCT	Male Wistar rats (*n* = 15)	12 wk	40 mg/kg.day BB extracts in 5 mL drinking water	Normal chow diet	Prevent capillary albumin filtration	[46]
6	RCT	Goto-Kakizaki (GK) rat (*n* = NS)	4 wk	BB decoction with rodent chow	powdered rodent chow	Improved mitochondrial respiratory and biogenesis parameters	[47]
(Human studies)							
7	DB, CO, RCT	T2DM Male volunteer subjects (*n* = 8)	24 h	A single capsule of 0 × 47 g BB extract (36% *w/w*)	NA	Decrease in the incremental AUC for both glucose and insulin without alterations in GLP1, glucagon, amylin, and anti-inflammatory peptides	[48]
8	CO, DB, RCT design	Obese/Overweight/diabetic men and women (*n* = 16)	3 wk	3 × 0.47 g of Mirtoselect capsules per day, a standardized BB extract (36% *w/w*) anthocyanins)	NA	Reduced activity of digestion enzymes without alterations in anti-inflammatory markers, vascular health markers and reducing capacity	[49]
9	RCT	Healthy men and women (*n* = 9)	1 d	10% BB in fermented drink up to 300 g/day	White bread	Lower the insulin response than glycemic response	[50]
10	RCT	Healthy men and women (*n* = 62)	4 wk	BB juice 330 mL/day	NA	Anti-inflammatory	[51]
11	Parallel RCT	Healthy men (*n* = 40)	8 wk	Fresh BB 100 g/day of BB	NA	Increased intestinal bioavailability of antioxidative and antidiabetic compounds	[52]
12	RCT	Healthy men and women (*n* = 27)	8 wk	Fresh BB 400 g/day	NA	Reduction in the low-grade inflammation with different cytoplasmic ribosomal proteins, Toll-like receptor, and B-cell receptor signaling pathways	[53]
(I) Cranberries (CrB) (Animal studies)							
1	RCT	36 C57Bl/6J male mice	8 wk	CrB extracts 200 mg/kg BW on daily basis by gavage	HFD (65% lipids, 15% proteins and 20% carbohydrates)	Attenuated HFD-induced obesity, TC and TG accumulation, oxidative stress, with improvements in glycemic response, insulin sensitivity, HOMAIR, alleviate intestinal inflammation	[54]
2	RCT	Male Fischer rats (*n* = 24)	16 m	2% whole CrB powder standard NIH-31 standard rodent chow available *ad libitum*	NA	Increased β-cell glucose responsiveness; age related decline in in basal plasma insulin concentrations was delayed by cranberry	[55]
Human studies							
3	CO, RCT	Obese participants (*n* = 25)	2–4 h	Cranberries (40 g)	HF breakfast (70 g fat, 974 kcal)	Improved postprandial glycemic control, reduction in lipid oxidation products and inflammatory cytokines	[56]
4	PC, DB, RCT	T2DM men and women (*n* = 30)	12 wk	3 capsules of CrB extract/day (1 capsule = 500 mg)	NA	Decrease in the TC:HDLC ratio	[57]
5	single CO RCT	Healthy men and women (*n* = 12)	OTCS	Dextrose sweetened normal calorie CrB juice (NCCBJ; 27% CBJ, *v/v*; 130 Cal/240 mL) and low-calorie CrB juice (LCCBJ; 27%, v/v CrBJ;19 Cal/240 mL)	5 g Vanilla Crisp Power Bar (contained 230 Cal, 2.5 g total fat, 3 g dietary fiber, 20 g sugars, 22 g other carbo-hydrates, and 9 g protein	Improved metabolic response towards glucose	[58]
6	RCT	Non-diabetic men and women (*n* = 187)	OTCS	low-calorie 27% CrBJ (19 Cal/240 mL); normal-calorie 27%CrBJ (140 Cal/240 mL) at weight-adjusted serving size (480 mL/70 kg)	NA	Improved glycemic response	[59]
7	RCT	T2DM men and women (*n* = 13)	OTCS	Raw CrB (55 g, 21 cal, 1 g fiber); Sweetened dried CrB (40 g, 138 cal, 2.1 g fiber); Sweetened dried CrB containing less sugar (SDC-LS, 40 g, 113 cal, 1.8 g fiber + 10 g polydextrose)	White bread (57 g, 160 cal, 1 g fiber)	favorable glycemic and insulinemic response	[60]
8	CS Nutrition Examination Survey (*n* = 10 891)	Healthy men and women	2 days	Average 2-day CrBJ intake 158 to 404 mL	Routine diet	Lowered the weight-gain, TC, and proinflammatory serum CRP levels	[61]
9	DB, CO, RCT	Healthy men and women (*n* = 12)	OTCS	37·5 g of CrB in addition to 37.5 g × 3 of other berries (bilberries, strawberries, blueberries) + 35 g added sugar	NA	Hypoglycemic and hypo-insulinemic	[62]
10	Parallel RCT	Diabetic men and women (*n* = 48)	8 wk	200 × 2 mL RCCJ was enriched with omega-3 fatty acid (180 mg EPA + 120 mg DHA) on daily basis	usual diet and physical activity during the study	Anti-dyslipidemic and hypoglycemic	[63]
11	Parallel DB RCT	T2DM male patients (*n* = 58)	12 wk	1 cup (240 mL) CrB juice daily	NA	Antiglycation, antidiabetic, reducing CVD risk factors in T2DM male patients	[64]
12	Parallel DB, PC RCT	Healthy men and women (*n* = 56)	8 wk	480 mL (80 kcal) whole CrB juice daily	Complete diets in addition to Background diets consisted of typical American foods (HFD) and 3–5 servings of fruits or vegetables daily (328– 618 g/d depending on energy intake)	Anti-dyslipidemic, hypoglycemic, improved HOAM-IR	[65]
13	RCT	Patients with metabolic syndrome (*n* = 55)	60 d	0·7 L/day (J (20 kcal) of reduced-energy CrB juice containing 66 mg proanthocyanidins; total phenolics of 104 and 0·12 mg folic acid	NA	An increase in adiponectin and folic acid and a decrease in homocysteine, decreased lipoperoxidation and protein oxidation levels	[66]
14	CO, DB	Obese yet healthy men (*n* = 35)	4 wk	500 mL CrB juice/day	NA	Improved augmentation in obese men	[67]
15	DB, CO	Obese men (*n* = 30)	12 wk	Increasing doses of low-calories CrBJ during three successive periods of 4 wk (wk 1–4: 125 mL/day, wk 5–8: 250 mL/day, and wk 9–12: 500 mL/day)	NA	Improved antioxidative defense system	[68]
16	DB, CO	Obese men (*n* = 30)	12 wk	Increasing doses of low-calories CrBJ during three successive periods of 4 wk (wk 1–4: 125 mL/day, wk 5–8: 250 mL/day, and wk 9–12: 500 mL/day)	NA	Decrease in plasma OxLDL, intercellular adhesion molecule-1 (ICAM-1), vascular cell adhesion molecule-1 (VCAM-1) and E-selectin concentrations	[69]
17	CO, DB	Abdominally obese men (*n* = 30)	12 wk	Increasing doses of low-calories CrBJ during three successive periods of 4 wk (wk 1–4: 125 mL/day, wk 5–8: 250 mL/day, and wk 9–12: 500 mL/day)	NA	Increased plasma HDL-cholesterol concentrations	[70]
18	RCT	Healthy men (*n* = 21)	2 wk	CrBJ 7 mL/kg.BW.day	NA	Reduction in plasma OxLDL levels and Improved antioxidative defense system	[71]
(I) Raspberries (RB) (Animal studies)							
1	RCT	Weanling male Syrian golden hamsters	12 wk	RBJ 275 mL/day (1 mL = 0.6 g of berries)	semi-purified hyperlipidic diet (0.5% cholesterol and 15% lard)	Hypo-cholestrolemic and antioxidative	[72]
2	RCT	Male Wistar rats (*n* = 30)	10 d	Dose of ellagitannins enriched RB extracts equivalent to daily consumption of 125 g of fresh fruit by a human healthy adult of 70 kg (i.e., 20 mg/kg BW.day orally)	NA	Protection from the ethanol induced oxidative stress and inflammatory biomarkers	[73]
3	RCT	male Lewis rats (*n* = 24)	30 d	RB extracts at 30–120 mg/kg.BW	NA	Inhibition of inflammation, pannus formation, cartilage damage, and bone resorption	[74]
4	RCT	CD1 male mice (*n* = 36)	4 wk	RB infusion by gavage (100 mg/kg BW.day)	NA	Improved antioxidative defense system	[75]
5	RCT	obese diabetic (db/db) mice (*n* = 30)	8 wk	5.3% RB supplementation along agar-based diet finally containing polyphenolics (963 mg extractable GAE/kg agar-based diet)	agar-based diet	Hyper-cholestrolemic and diabetes-induced oxidative stress	[76]
6	RCT	Male Zucker Fatty rats (*n* = NS)	12 wk	20 g of diet per day containing RB (2% red raspberry F/D powder)	NA	Upregulation of the expression of myocardial adiponectin receptor 1 and apolipoprotein E, improving the plasma cholesterol and triglyceride homeostasis	[77]
7	RCT	Male Wistar rats (*n* = 42)	5 d	313 g whole RB with/without *Lactobacillus plantarum* HEAL19 (HEAL19 at 10^9^ cfu) per day with diet	Normal chow diet	Increased intestinal SCFA load and anti-inflammatory	[78]
8	RCT	Male F-344 rats (*n* = NS)	6 wk	AIN-76A diet containing either 5% whole BRB powder, 0.2% BRB anthocyanins, or 2.25% of the residue fraction provided ad libitum	NA	Anti-dysbiosis, anti-inflammatory, anti-obesity	[79]
9	RCT	Male db/db mice with C57BL/6J Background (*n* = 48)	8 wk	150 mg/kg.BW.day per mice RB derived pelargonidin-3-*O*-glucoside	NA	Hypoglycemic, anti-inflammatory, anti-obesity	[80]
10	RCT	Specific-pathogen free C57BL/6 mice (*n* = 20)	7 wk	AIN-76A diet with 10% black raspberry powder provided ad libitum	NA	Hypo-glycemic, anti-metabolic syndromic	[81]
11	RCT	Male db/db mice (*n* = 30)	8 wk	10% F/D RB in a isocaloric standard diet	Isocaloric standard diet	Hypo-cholestrolemic, antioxidative, improved insulin sensitivity	[82]
12	RCT	C57BL/6J mice (*n* = NS)	10 wk	Energy-containing RB foods (juice and puree concentrate and whole fruit powder) containing 10% raspberry and HFD supplemented with 0.2% (*w/w*) RB extract provided ad libitum	HFD (45% energy from fat) + high-carbohydrate food (35% energy from starch)	Anti-obesity and antidiabetic	[83]
13	RCT	C57BL/6J, C57BL/Ks db/db, and db/+ male mice (*n* = NS)	8 wk	0.2% Cyanidin 3-glucoside in HFD	HFD (58% of calories from coconut hydrogenated fat)	Anti-obesity, anti-inflammatory, improvement in the insulin sensitivity	[84]
14	RCT	Male Sprague Dawley rats (*n* = 40)	8 wk	Application of RB derived EA (0.1–10 mg/mL) on ischemic stomach (1.5 mL/100 g.BW) in an in an ex vivo chamber	NA	Gastric protective action against gastric lesions induced by NH_4_OH, due to anti-oxidative activity of EA	[85]
15	RCT	Male Wistar rats (*n* = 22)	4 wk	Oral administration of 10–20 mg/kg.BW of RB derived elagic acid	NA	Anti-inflammatory and anti-oxidative	[86]
16	RCT	Male Wistar rats AMPKα1−/− (*n* = 12)	10 wk	5% supplementation of RB extracts (contains polyphenols at ~11 g gallic acid equivalent (GAE)/kg of DW) along HFD	HFD (60% from fat)	reduced ectopic lipid storage, alleviated inflammation responses, improved whole-body insulin sensitivity, and promoted mitochondrial biogenesis	[87]
17	RCT	Male mice (C57BL/6) (*n* = 40)	12 wk	5% F/D RB powder in HFD provided ad libitum	HFD (60% energy from fat)	Anti-dyslipidemic, hypoglycemic	[88]
18	RCT	Male KK-Ay mice (*n* = NS)	5 weeks	Cyanidin 3-glucoside 2 g/kg.BW.day in the normal chow diet	NA	Anti-obesity, anti-inflammatory, improvement in the insulin sensitivity	[89]
19	RCT	Male mice (C57BL/6) (*n* = 40)	12 wk	3% RB seed floor (equivalent to 0.03% ellagic acid) in HFD and HFD + High-sucrose diet	HFD (41% energy from fat) HFD + High-sucrose diet (37% energy from sucrose)	Anti-dyslipidemic, hypoglycemic, attenuated hepatic ER and oxidative stresses, as well as adipocyte inflammation	[90]
Human studies							
20	PC, CO, RCT	Healthy men and women (*n* = 20)	4 wk	High-carbohydrate bars (120–123 g) containing freeze-dried black RB (10% (LOW-Rasp) or 20% (HIGH-Rasp)), One bar each day after overnight fasting.	macronutrient-matched high-carbohydrate cereal bars (45% total sugars)		[91]
21	RCT	Healthy men and women (*n* = 12)	NS	100 g RB along the designated diet	High-carbohydrate food in the form of pancakes (50 g available carbohydrate from 333 kcal pancake)	Alter postprandial hyperglycemia to sustainable glycemic response	[92]
22	3 randomized, controlled, CO,	Healthy women (*n* = 13–20)	OTCS	150 g whole berries puree along each meal study 1: white bread + strawberries, bilberries, or lingonberries study 2: white bread + h raspberries, cloudberries, or chokeberries study 3: white bread or rye bread + mix berries consisting of equal amounts of strawberries, bilberries, cranberries, and blackcurrants	White bread or rye bread with 50 g available starch	Reduced the postprandial insulin response, improved the glycemic profile, improved postprandial glucose metabolism.	[93]
23	CO, RCT	T2DM men and women (*n* = NS)	12 wk	250 g frozen red raspberries puree with each breakfast	NA	Anti-dyslipidemic, anti-inflammatory, anti-obesity	[94]
	(I) Mulberries (MBs) (Animal studies)						
1	Randomized block design	Male C57BL/6 mice (*n* = 60)	8 wk	MB anthocyanins at 200 mg/kg HFD provided ad libitum	HFD (45% kcal from fat)	Anti-dyslipidemia, anti-inflammatory, anti-obesity	[95]
2	RCT	Male db/db mice with C57BL6/J genetic background (*n* = 50)	8 wk	MB fruit extracts 50 and 125 mg/kg BW every day orally by gavage	NA	Antioxidative and hypoglycemic	[96]
3	RCT	male adult Wistar rats (*n* = 70)	6 wk	MB fruit wine 400 mL/70 kg of body weight daily	NA	Antioxidative and hypoglycemic	[97]
4	RCT	male Sprague–Dawley rats (*n* = 50)	8 wk	MB fruit derived cyanidin-3-*O*-β-D-glucopyranoside (10 mg/kg.BW. daily) orally by gavage	NA	Antidiabetic cystopathy	[98]
5	RCT	Adult diabetic male Wistar rats (*n* = 12)	6 wk	MB polysaccharides (200 mg/kg.BW daily)	HFD	Improved oral glucose tolerance/insulin resistance, bioactivities of superoxide dismutase (SOD), catalase (CAT) and glutathione peroxidase (GPx), were increased	[99]
6	RCT	Male Gold Syrian hamsters (*n* = NS)	12 wk	Water extracts of MB fruit at 1–2% (*w/w*) in HFD provided ad libitum	HFD (1% cholesterol and 10% corn oil)	Hypolipidemic	[100]
7	RCT	Male C57BL/6 mice (*n* = 48)	12 wk	Anthocyanin from MB of 40–200 mg/kg of HFD	HFD (45% kcal from fat)	Inhibit body weight gain, reduce the resistance to insulin, lower the size of adipocytes, attenuate lipid accumulation and decrease the leptin secretion.	[101]
8	RCT	Male Syrian golden hamsters (*n* = 32)	12 wk	Water extracts of MB fruit at 0.5–2% (w/w) in HFD provided ad libitum	HFD (10% corn oil + 0.1% cholesterol)	Anti-obese and hypolipidemic effects	[102]
9	RCT	Male Wistar rats (*n* = 32)	4 wk	5–10% (w/w) mulberry fruit polysaccharide fractions in HFD provided ad libitum	HFD (10% lard, 1% cholesterol, 0.5% sodium cholate, and 88.5% commercial diet)	Hypolipidemic and improved the enzymatic antioxidant system	[103]
10	RCT	Male C57BL/6 mice (*n* = 48)	6 wk	0.5–2% (w/w) water extracts of MB fruit in high-fat (35% kcal from fat) ethanol rich liquid diet (36%kcal from ethanol) provided ad libitum	high-fat (35% kcal from fat) ethanol rich liquid diet (36%kcal from ethanol)	Anti-obesity, hypoglycemic, antioxidative, anti-inflammatory	[104]
11	RCT	Male Sprague-Dawley rats (*n* = 40)	10 wk	MB fruit extracts 100 or 200 mg/kg.BW.day	HFD (1% cholesterol, 18% lipid (lard), 40% sucrose)	Anti-dyslipidemic, antioxidative, ameliorates nonalcoholic fatty liver disease (NAFLD)	[105]
12	RCT	Female Wistar rats (*n* = 48)	20 wk	Microencapsulated 50 to 250 mg/kg.BW.day mulberry fruit extracts (microencapsulated) with HCHF	High-carbohydrate high-fat (HCHF) diet which contained total energy around 4.62 kcal/g (fat 31.54%, protein 20.25%, and carbohydrate 48.21%).	Anti-inflammatory, antioxidative, improved metabolic syndrome	[106]
13	RCT	Male, C57BL/6J mice (*n* = 12)	13 wk	20% MB powder in HFD provided ad libitum	HFD, 60% calories from fat	Anti-obesity, antidiabetic, increase of *Bacteroidetes/Firmicutes* ratio	[107]
14	RCT	db/m mice (*n* = 50)	NS	MB fruit polysaccharide fractions (200–800 mg/kg.BW)	NA	Improved antioxidant enzymatic defense system, antihyperglycemic and antihyperlipidemic effects	[108]
15	RCT	Male C57BL6/J genetic background (db/db) mice (*n* = 60)	8 wk	Mulberry fruit extract 25–250 mg/kg BW daily	NA	Upregulation of gluconeogenesis pathway	[109]
16	RCT	Adult diabetic male Wistar (*n* = 40)	7 wk	MB fruit polysaccharide fractions MFP50 and MFP90 (400 mg/kg.BW)	HFD	Antihyperglycemic and antihyperlipidemic effects	[110]
	(I) Lingonberries (LB) (Animal studies)						
1	RCT	Male C57BL/6 mice (*n* = NS)	8 wk	LB extracts (125, 250, and 500 mg/kg) in HFD provided ad libitum	HFD (35% fat, 20% protein, and 36.5% carbohydrate)	Attenuates hepatic steatosis hyperglycemia, hyperlipidemia. Improves insulin signaling	[111]
2	RCT	SHR rats (*n* = NS)	8 wk	Cold-compressed LB juice provided ad libitum	NA	Reduced hypertension and pro-inflammatory markers	[112]
3	RCT	Male C57BL/6JBomTac mice (*n* = 120)	13 wk	20% (*w/w*) F/D LB in HFD provided ad libitum	HFD (45 kcal% fat)	Significantly reduced body fat, lipid accumulation, and plasma levels of the inflammatory marker PAI-1, as well as mediated positive effects on glucose metabolism homeostasis.	[113]
4	RCT	Male C57BL/6JBomTac mice (*n* = NS)	11 wk	20% (*w/w*) F/D LB in HFD provided ad libitum	HFD (45 kcal% fat)	Reduced plasma levels of markers of endotoxemia and inflammation	[114]
5	RCT	Male Apoe-/- mice (*n* = 35)	8 wk	44% lingonberry + HFD	HFD (38 kcal% fat)	Decreased triglyceridemia and reduced atherosclerosis	[115]
6	RCT	Male C57BL/6JBomTac mice (*n* = NS)	11 wk	20% (*w/w*) F/D LB in HFD provided ad libitum	HFD (45 kcal% fat)	Improvement in glycaemia, reduction in inflammation and hepatic steatosis	[116]
7	RCT	C57BL/6JBomTac (*n* = NS)	13 wk	20% (*w/w*) freeze-dried LB + blackcurrants, bilberries or açai berry in HFD provided ad libitum	HFD (45 kcal% fat)	Downregulation of inflammatory pathways, NF-κB, STAT3 and mTOR as possible targets for antidiabetic therapy	[117]
8	RCT	Male ApoE−/− mice (*n* = 50)	8 wk	Two LB polysaccharide fractions 15–60 g/kg BW with HFD daily	HFD (38 kcal% fat)	Hypoglycemic, hypolipidemic, altered caecal microbiota composition	[118]
Human studies							
9	RCT	Scandinavian type 2 diabetes patients (*n* = 30)	12 wk	Recommended daily intake of LB/berries/fruits	Okinawan-based Nordic diet of about 1,900 kcal/day	Improved metabolic and anthropometric parameters	[119]
10	CO, DB, RCT	Healthy normal-weight nonsmoking men (*n* = NS)	6 d	Glycemic diet + 40 g lingonberry powder Lipemic diet + 60 g lingonberry powder	Glycemic diet: 200 g yoghurt (lactose-free and fat-free non-flavored natural yoghurt + 50 g glucose) Lipemic diet: 200 g Yoghurt (lactose-free and fat-free non-flavored natural yoghurt + 35 canola oil)	Nullified the glycemic effect of the sugars present in the meals without affecting the postprandial lipemic response	[120]
11	CO, DB, RCT	13 Healthy, over-weight, non-smoking male and female volunteers	Single meal challenge	100 g lingonberry	Hyperlipidic and hypercaloric meals (38 kcal% fat)	Reduced glycemic response, rarified the increase of cholesterolemia	[121]
12	RCT	Normal, healthy subjects (*n* = 9)	12 wk	LB polysaccharides + fibers (2 g/Kg of oat bread)	Oat bread	In reduced glucose and C-peptide response	[122]
13	SB, CO, RCT	Healthy women volunteers (*n* = 20)	2-h meal tests	Diet 1: 150 g whole LB puree containing 35 g sucrose per day Diet 2: 300 mL LB nectar (equal to 150 g fresh berries) containing 35 g sucrose	NA	Optimized postprandial metabolic responses to sucrose with delayed digestion and absorption of sucrose/glucose	[123]
14	RCT	Healthy non-smoking males (*n* = 14)	2-h meal tests	60 g of LB juice press residue corresponding to 270 g of fresh LB with standard diet	Standard diet: white wheat bread, cucumber, water, and a banana	Gut microfloral metabolism of polyphenols resulting in increased levels of hippuric acid and 4-hydroxyhippuric acid	[124]
(I) Blackberries (BBR) (Animal studies)							
1	RCT	Male Wistar rats (*n* = 32)	5 wk	Anthocyanin-enriched fraction (AF) and Ellagitannin-enriched fraction (EF) equivalent to (4 mg cyanidin eq/kg BW) and 2.68 mg EA eq/kg BW respectively	NA	Reinforce the antioxidative defense system and lipid oxidation markers	[125]
2	RCT	C57BL/6 mice (*n* = 60)	12 wk	BBR extracts at 200 mg/kg food BBR extracts: cyanidin-3-glucoside (51.24%), cyanidin-3-rutinoside (42.31%), and peonidin-3glucoside (6.91%)	HFD (45% kcal from fat)	Anti-inflammatory, anti-hypertensive, anti-hypercholesterolemia, antioxidative	[126]
3	RCT	Male DIO C57BL/6J mice (*n* = 40)	12 wk	6.3%, (w/w) BBR extracts in HFD provided ad libitum	HFD (45% kcal from fat)	Anti-obesity, Anti-inflammatory, anti-hypertensive,	[127]
4	RCT	Male Wistar rats (*n* = 24)	17 wk	25 mg/kg.BW BBR extracts in HFD provided ad libitum	HFD (45% kcal from fat)	Anti-obesity, anti-inflammatory, anti-dyslipidemic	[128]
5	RCT	Male diabetic Sprague-Dawley rats (*n* = 40)	40 d	Microfiltrated 12.5–25% BBR juices		Reduced glycaemia (−10.4%), TG (−4.6%) and TC (21.0%), lipid peroxidation, attenuation of oxidative stress	[129]
6	RCT	Male Wistar strain rats (*n* = 40)	4 wk	Normal standard diet with 0.98% BBR polyphenols and 6% BBR fiber	Normal chow diet	Anti-inflammatory and anti-dyslipidemic	[130]
7	RCT	Female obese (BKS(D)-Leprdb/J72) and lean (C57BL/6J) mice (*n* = 24)	10 wk	Aged or fresh BBR supplemented at 10% (w/w) of diet provided ad libitum	Normal chow diet	Increased in total beneficial bacterial population	[131]
8	RCT	Male C57BL/6J mice (*n* = 72)	10 wk	Alcohol-free blueberry–blackberry fermented beverage (AFFB) a) AFFB [70% blackberry and 30% blueberry, 8.4 mg cyanidin-3-*O*-glucoside (C3G) eq./kg.BW)/day]; (b) dose 0.1 × ostamberlite extract (PAE), 1.1 mgC3G eq./kg BW/day; (c) dose 1 × PAE, 9.0 mg C3G eq./kg BW/day; (d) dose 2 × PAE, 18.9 mg C3G eq./kg BW/day	HFD (60.3% fat, 21.3% carbohydrate and 18.4% protein)	Reduced percent fat mass, mean adipocyte diameters, epididymal fat pad weights, and plasma TG and TC.	[22]
	Human studies						
9	RCT	Diabetic and obese men and women (*n* = 152)	1 wk	Consumption of daily recommended amount of low glycemic index fruit (0.7–1.4 servings/day)	NA	Anti-dyslipidemic	[132]
10	open, single-center RCT	Healthy human subjects (*n* = 6)	4 h	200 mL of BBR juice equivalent to 400 mg of cyanidin equivalent/50 kg of body weight	NA	Improved plasma and urine antioxidant system	[133]
11	RCT	Dyslipidemic patients (*n* = 72)	8 wk	300 mL of BBR juice (equivalent to 316 mg/100 g polyphenols) of BBR with pulp every day	NA	Increased apo A-1 and HDL-C along reduction in apo B and hsCRP	[134]
(I) Strawberries (SB) (Animal studies)							
1	RCT	Diabetic male albino Wistar rats (*n* = 36)	4 wk	Aqueous, alcoholic and hydro-alcoholic SB extract (2 g/kg b.w.day	NA	Reduced expression level of genes involving glucose, lipid metabolism with improvement in glucose metabolism and liver function	[135]
2	RCT	Male Wistar rats (*n* = 20)	12 wk	HFD supplemented with 0.2% irradiated/non-irradiated SB extracts	HFD (47.5% kcal from fat)	Reduction in the oxidative damage in brain and peripheral tissues	[136]
3	RCT	Male C57BL/6J mice (*n* = 36)	24 wk	HFD supplemented with 2.6% freeze-dried SB	HFD containing approximately 20% higher in energy density compared to the low-fat diets	Reduction in the HFD led increase of FBS, adhesion molecule-1, leptin, E-selectin, resistin, and plasminogen activator protein-1	[137]
4	RCT	Male Wistar rats (*n* = 48)	8 wk	Supplementation of the diet with a 6% w/w (equivalent to a 5 g/kg 65 BW dose) of a F/D SB-BlB (5:1) powder (FDSB)	High-fat-sucrose diet (D12451, Research Diet)	Anti-obesogenic and anti-inflammatory effects	[138]
5	RCT	Male Wistar rats (*n* = 24)	16 wk	AIN93-modified diet with lyophilized SB extract at 10 g/kg of diet	AIN93-modified diet	Improvement of oxidative stress biomarkers, mitochondrial performance, antioxidant enzyme activities, reduction of DNA damage and ROS concentration	[139]
6	RCT	Male Wistar rats (*n* = 20)	12 wk	Supplementation of 0.2% SB	HFD (47.5% calories from fat)	Antioxidative, anti-stress	[140]
7	RCT	German Landrace pigs (*n* = 48)	4 wk	205–745 g of SB with normal feed per day	Linseed oil (15 g/day) enriched feed	Anti-stress and antioxidative	[22]
8	RCT	db/db mice homozygous for the diabetes spontaneous mutation (Leprdb) with C57BL/6J background (*n* = 24)	10 wk	2.35% F/D SB powder in the diet pellets (*w/w*) (equivalent to two human servings of SB i.e., ~160 g SB)	NA	Increased *Bacteriodetes* to *Firmicutes* ratio	[141]
9	RCT	Male CD-1 mice (*n* = 60)	8 wk	5% (*w/w*) of diet freeze-dried whole SB powder	AIN93G diet	Increased *Bacteriodetes* to *Firmicutes* ratio	[142]
	(Human studies)						
10	DB, RCT, parallel study	Insulin resistant and obese males and females (*n* = 41)	6 wk	Beverage containing 1·84 g of a mixture of dry SB and CrB providing 333 mg of polyphenols on daily basis (also equivalent to 112 g consumption of fresh berry fruit)	NA	Improved insulin sensitivity and release	[143]
11	CO, SB, PC, RCT	Hyperlipidemic men and women (*n* = 24)	12 wk	SB beverage containing 10 g/serving of freeze-dry SB powder providing 338 mg of polyphenols daily (also equivalent to 110 g consumption of fresh berry fruit)	HFD consisting of typical breakfast food items (i.e., bagel, cream cheese, whole milk, egg, margarine, cantaloupe)	Reduced postprandial lipemia and oxidative stress markers	[144]
12	CO, RCT	Healthy males and females (*n* = 30)	5 d	20 g of five types SB jams each with sugar of different glycemic index	60 g white bread slice	Non-significant reduction in the postprandial glucose level	[145]
13	CO DB RCT	Healthy males and females (*n* = 16)	3 wk	60 g of three types SB jams each with sugar of different glycemic index and polyphenolic contents		Strawberry jam with high sugar level produced less levels of FFA.	[146]
14	DB RCT	T2DM males and female subjects (*n* = 36)	6 wk	Two cups of F/D SB beverage containing 25 g × 2 = 50 g	NA	Reduction in LDL-C and LDL-C/TC and LDL-C/HDL-C ratio	[147]
15	SB, CO parallel, RCT	Obese and overweight men and women (*n* = 24)	6 wk	SB beverage containing 10 g/serving of freeze-dry SB powder providing 96 mg of polyphenols on daily (also equivalent to 100 g consumption of fresh berry fruit)	High-carbohydrate-fat diet	Attenuation of diet-induced inflammatory markers	[148]
16	Single-center, CO, SB, PC,	Men and women (*n* = 26)	OTCS	SB Milk based beverage containing 10 g/305 mL of F/D SB powder	high-carbohydrate, moderate-fat meal (HCFM)	Reduced postprandial insulin and inflammatory response	[149]
17	Four-arm, SB, PC, CO, RCT	Males and females with insulin resistance (*n* = 23)	NS	SB milkshake containing 10–40 g freeze-dried SB powder where 10 g freeze dried powder = 110 g fresh strawberries	Standard western type meal	Reduced lipid oxidation and post-meal insulin demand	[150]
18	Observatory study	Healthy men and women (*n* = 247)	20 years	Dietary flavonoids intake (47–560 mg/day) from fruits and berries	-	Flavonoid Compounds in Driving Patterns of Microbial Community Assembly	[151]
19	RCT	Obese men and women (*n* = 66)	12 wk	SB beverage containing 25–50 g freeze-dry SB powder daily	HFD (50% calories from fat)	Increased the glutathione level, serum catalase activity, and plasma antioxidant capacity	[152]
20	DB RCT	T2DM patients (*n* = 40)	6 wk	50 g of freeze-dried SB powder (equivalent to 500 g fresh strawberries) each day	NA	Reduction in the markers of lipid peroxidation (MDA), inflammatory markers (CRP). Reducing trend in HbA1c.	[153]
(I) Goji berries (GB) (Animal studies)							
1	RCT	Alloxan-induced hyperglycemic/hyperlipidemic adult rabbits (*n* = 35) and male mice (*n* = 24)	10 d	Water decoction (0.25 g/kg BW day), crude GB polysaccharides (10 mg/kg BW day), and purified GB polysaccharides (10 mg/kg BW day)	NA	Hypoglycemic and hypolipidemic effect with increased plasma antioxidant capacity	[154]
2	RCT	Male Wistar rats (*n* = 70)	8 wk	Ethanolic and aqueous GB extracts at 50 mg/kg b.w. or 100 mg/kg BW daily	HFD	Significantly reduced liver damage and oxidative changes	[155]
3	RCT	Diabetic male mice of original Kun-ming strain (*n* = NS)	4 wk	GB polysaccharides (20–40 mg/kg BW day) orally	NA	Hypoglycemic and hypolipidemic	[156]
4	randomized block design	Obese male Sprague-Dawley rats (*n* = 60)	8 wk	GB anthocyanins at 50–200 mg/kg BW.day	HFD	Reduced body-weight-gain with anti-inflammatory properties	[157]
5	RCT	STZ-diabetic Male Wistar rats (*n* = NS)	8 wk	GB polysaccharides (10 mg/kg, BW.day)	NA	Increased antioxidative scavenging and antioxidant enzymes. Increased activity of protein kinase C (PKC)	[158]
6	RCT	STZ-induced diabetic Sprague-Dawley male rats (*n* = 60)	8 wk	Water decoction of GB (5 g/kg.BW.day)	NA	Protective effects in diabetic retinopathy	[159]
8	RCT	Male Wistar rats (*n* = 16)	4 wk	GB polysaccharides 10 mg/kg BW.day dissolved in physiological saline	High-fat-sucrose diet	hypoglycemic and improving hyperinsulinemia	[160]
9	RCT	Diabetic male C57BL/6J mice (*n* = 48)	7 wk	GB polysaccharides 100–500 mg/kg BW.day by gastric perfusion	HFD	Hypoglycemic effects with increased insulin-sensitizing, glucose metabolism, insulin secretion, and promoting pancreatic cell proliferation.	[26]
10	RCT	Swiss Albino rat (*n* = 30)	3 wk	Water-soluble polysaccharides (galactomannan) 250–500 mg/kg BW.day by oral gavage	NA	Hypolipidemic, reduced lipid oxidation, increased insulin-sensitizing and serum antioxidant level	[161]
11	RCT	Diabetic Wistar rats (*n* = 48)	8 wk	Water-soluble GB polysaccharides 250–500 mg/kg BW.day by oral gavage	HFD and HCD (12% protein, 5% fat, 67% carbohydrate, 5% cholesterol, and 5% other additives)	Reduced serum level of IL-2, IL-6, TNF-α, IFN-α, MCP-1, and ICAM-1 with increased activities of SOD and GSH-Px activities	[162]
12	RCT	Postnatal Royal College of Surgeons (RCS) rats (*n* = 60)	4 wk	Whole GB powder 1 mg/kg of per day	NA	Reduced Caspase-2 activity in experimental group at 25th post-neonatal day	[163]
13	RCT	Male IL-10-deficient mice (*n* = 14)	10 wk	Diet supplemented with 1% GB	Normal diet	Increased gut population of SCFA producing bacteria	[164]
Human studies							
14	RCT	Kunming mice of clean grade (*n* = 14)	2 wk	GBPS at a dose of 0.1 mL/10 g body weigh daily via intragastric administration	Normal diet	Increased gut population of SCFA producing bacteria, *Firmicutes*, *Akkermansia, Lactobacillus*, and *Prevotellaceae*	[165]
15	DB, PC, RCT	Healthy males and females (*n* = 50)	30 d	Intake of 120 mL of GB juice (equivalent to 1632 mg/ daily serving (120 mL) of goji berry polyphenols	Traditional Chinese diet rich in carbohydrate	Increased serum levels of glutathione peroxidase (GSH-Px) and superoxide dismutase (SOD) with reduced level of MDA	[166]
16	RCT	Metabolic syndrome patients (*n* = NS)	45 d	14 g of GB with meals	Normal diet	Reduction in transaminases, waist circumference with improvements in lipid profile, glutathione and catalase level.	[167]
17	RCT	Male and female C57BL/b6N mice (*n* = 56)	8 wk	GB polysaccharides (1–10 mg/kg BW day) orally	NA	Increased hepatic antioxidant enzymes, y inhibited cytochrome P450 2E1, nitric oxide metabolism and lipid peroxidation	[168]
18	DB, CO, RCT	healthy overweight men (*n* = NS)	Single meal challenge	meal containing 25 g of dried GB fruit	Ready-made meal with a fixed macronutrient composition (30–40% fat, 40–50% carbohydrates, and 13–16% proteins)	No-single-dose-effect on substrate oxidation and prospandial-energy-expenditure	[169]
(I) Acai berries (AB) (Animal studies)							
1	RCT	Male mice of the C57BL/6 strain (*n* = NS)	12 wk	AB seed extracts 300 mg/kg.BW.day by intragastric gavage	HFD (60% calorie from fat)	Reduced expressions of lipogenic proteins (SREBP-1c, pACC, ACC, HMG-CoA reductase) with increased expression of pAMPK, pACC/ACC, and cholesterol transporters (ABCG5 and ABCG8)	[170]
2	RCT	Zebrafish (*n* = 70)	5 wk	HC diet supplemented with10% w/w of AB puree powder	high cholesterol (HC) diet (47.5% crude protein, 6.5% crude fat, 4% cholesterol, 2.0% crude fiber, 10.5% crude ash)	Reduced oxidative markers with lipid lowering effects	[171]
3	RCT	Oxidatively damaged sod1/sod1 mutant strains *Drosophila melanogaster* (*n* = 120)	5-6d	AB supplemented sugar-yeast (SY) medium to a final concentration of 0.25%, 0.5%, 1% or 2% (w/v) of the food	SY medium	Increased transcript level of gluconeogenesis gene phosphoenolpyruvate carboxykinase (Pepck) with reduction in oxidative stress	[172]
4	RCT	ApoE-deficient (ApoE 2/2) male mice (*n* = 23)	12 wk	AIN-93M diet formulated to contain 2% F/D açai’ pulp + exercise in progressive treadmill for 30 min daily at a speed of 12 m/min, 0% incline	AIN-93M diet	Hepatic superoxide dismutase activity, mRNA expression of monocyte chemotactic protein-1, percentages of hepatic lipid droplets	[173]
5	RCT	STZ-induced diabetic Male Wistar rats (*n* = NS)	45 d	AB seed extracts 200 mg/kg.BW.day in drinking water	NA	Reduced oxidative damage by reducing the expression of caspase-3, IL-6, TNF-α and MCP-1	[174]
6	RCT	Female Fischer rats (*n* = 32)	6 wk	Hypercholesterolemic diet (25% soy oil and 1% cholesterol) supplemented with 2% AB (dry wt/wt)	Hypercholesterolemic diet (25% soy oil and 1% cholesterol)	Reduced expression of cholesterol biosynthesis genes HMG CoA-R, EBP-2, ApoB100, LDL-R, ABCG8, and CYP7A1	[175]
7	RCT	STZ-induced diabetic Male Wistar rats (*n* = 40)	9 wk	AB seed extracts 200 mg/kg.BW.day by intragastric gavage	HFD (55% calorie from fat)	Hypoglycemic and hypolipidemic with reduced expression of TNF-α and activating the insulin-signaling pathway in muscle and adipose tissue	[176]
8	RCT	Diabetic female Fisher rats (*n* = NS)	30 d	Standard AIN-93 diet supplemented with 2% (*w/w*) AB pulp	AIN-93	Modulate ROS production by neutrophils and improve the liver oxidant/antioxidant balance	[177]
	(Human studies)						
9	CO, DB, RCT	Overweight healthy males (*n* = 23)	Single day meal challenge	Frozen AB pulp (150 g) was prepared in a smoothie with 50 g banana	50 g banana and matched for fat with 1.5 g hexadecanoic acid [palmitic acid (16:0)] and 8.5 g sunflower oil [30% (9Z)-Octadec-9-enoic acid (oleic acid [18:1]), 60% (9Z,12Z)-9,12-Octadecadienoic acid (linoleic acid [18:2]), and 10% palmitic acid (16:0)],	Lower incremental area under the curve (iAUC) for total peroxide oxidative status after açai and increased the iAUC for insulin	[178]
10	RCT	Male Swiss mice (*n* = 32)	12 wk	A single daily dose freeze-dried AB pulp (3 g/kg) via gavage	HFD (32% lard and 1% cholesterol)	Attenuated hepatic steatosis and reduced lipid accumulation	[179]
11	four-way CO	Healthy men and women (*n* = 11)	Single dose study	100% clarified AB juice/pulp 7 mL/kg BW of each study	NA	Increased plasma antioxidant capacity without affecting generation of reactive oxygen species, and uric acid concentrations in plasma	[180]
12	open label pilot study	Overweight adults (*n* = 10)	30 d	Intake of 100 g AB pulp twice daily	NA	Postprandial increase in the AUC of plasma glucose with reduced TC, LDL-C, and LDL-C/HDL-C	[181]
	(I) Chokeberries (CB) (Animal studies)						
1	RCT	C57BL/6JmsSlc and KK-Ay male mice (N = 10, EACH GROUP)	4 wk	CB provided ad libitum	Normal chow diet	Duction of glucose-dependent insulinotropicpolypeptide (GIP) level	[182]
2	RCT	STZ-induced-diabetic-male ICR mice (*n* = 32)	4 wk	CB extract (10–100 mg/kg.BW) daily administered orally	NA	Hypoglycemic, hypolipidemic, antioxidative	[183]
3	RCT	C57BL/6N mice (*n* = 20)	12 wk	CB powder dissolved in water (50 mg/kg daily)	HFD (60 kcal% Lard)	Reduced the body and liver weight, lipid accumulation, PPARγ2, FAS, hepatic TG and leptin. Serum transaminases, indicators for liver antioxidant capacity were significantly increased.	[184]
4	RCT	Male C57BL/6J (*n* = 60)	8 wk	CB extracts (100 mg/kg.BW) dissolved in 0.5% carboxymethyl cellulose	HFD (containing 60% kcal fat)	Attenuated weight-gain, increase in serum TG, TC, LDL-C and better glucose tolerance	[185]
5	RCT	Male Wistar rats (*n* = NS)	6 wk	Aronia melanocarpa fruit juice (AMFJ) at doses 10 and 20 mL/kg	NA	Hypoglycemic, hypolipidemic	[186]
6	RCT	Polish Merino lambs (*n* = 24)	12 wk	150-300 g of chokeberry pomace per each kg of the complete feed mixture	Complete feed mixture	Hypoglycemic, hypolipidemic	[187]
7	RCT	Middle-aged non-medicated subjects with MS (*n* = 38) an healthy volunteers (*n* = 14)	8 wk	CB extracts 100 mg/kg.BW three times daily	NA	Beneficial changes in lipid profile, coagulation parameters, inhibition of platelet aggregation	[188]
8	RCT	Male Wistar rats (*n* = 24)	4 wk	Diet was supplemented by the extract from CB fruits (0.2% W/W) added at the expense of corn starch	Standard casein diet enriched with 0.5% of cholesterol. Exp group: the diets were modified by 8% of lard and 65% of fructose added at the expense of soybean oil and maize starch,	Maltase and sucrase, e improvement of antioxidant status, cholesterol-lowering,	[189]
9	RCT	Male Wistar rats (*n* = NS)	4 wk	CB juice 10 mL/kg.BW.day	NA	Hypoglycemic, hypolipidemic, antioxidative	[190]
10	RCT	Male Wistar rats (*n* = 72)	8 wk	CB juice 50 mL/kg.BW.day	High-carbohydrate, high-fat + purple maize flour (HPM)	Reduced Inflammatory cell infiltration, visceral adiposity index, total body fat mass, improved glucose tolerance	[191]
11	RCT	Male Wistar rats (*n* = 36)	6 wk	CBE at 100 or 200 mg/kg BW.day	Fructose rich diet containing (g/kg diet): casein, 207; DL-methionine, 3·0; fructose, 600; lard, 50; cellulose, 79·8;	Elevated plasma adiponectin levels and inhibited plasma TNF-α and IL6. Increased in the expression level of glucose and lipid metabolizing genes	[192]
12	RCT	Male Wistar albino rats (*n* = 60)	4 wk	Standardized Aronia extract (SAE) 0.45 mL/kg.BW day) for 4 weeks	HFD (25% fat, 15% protein, 51% starch, and 5% fiber)	Reduced serum level of TC, TG, LDL-C, with increased serum levels of SFA and PUFA.	[193]
	(Human studies)						
13	CO open-label trial	T2DM patients (*n* = 35)	12 wk	Oral CB juice supplementation (150 mL/day, three times a day for 50 mL)	NA	Significantly improved the renal /hematological and lipid parameters (TG, TC, LDL-C, LDL-C/HDL-C) in diabetic patients	[194]
14	RCT	Healthy female volunteers (*n* = 29)	12 wk	100 mL of polyphenol-rich organic CB juice per day	NA	Reduced TBARS, pro-oxidantantioxidant balance, increase in paroxonase-1 activity	[195]
15	RCT	Apparently healthy women (*n* = 25)	12 wk	Consume 100 mL of polyphenol-rich organic CB juice daily	NA	Increased SOD and GPX activities, C22:6n-3, PUFAs, total PUFAs and unsaturation index and decrease in n-6:n-3 ratio	[196]
16	RCT	Healthy volunteers and 25 patients with metabolic syndrome (*n* = 22)	8 wk	CB extract (3 × 100 mg/day)	NA	Improvement in serum lipids, and oxidative status (GSH-Px, SOD, TBARS)	[197]
17	RCT	Healthy subjects (*n* = 33)	4 wk	Consume 200 mL of polyphenol-rich organic CB juice daily (containing 386 ± 9.7 mg of total phenolics expressed as gallic acid equivalents per 100 g)	NA	Positive effects on BP and lipid status in hypertensive subjects	[198]
18	RCT	Diabetic Wistar white male rats (*n* = 48)	16 wk	dose of polyphenols extracts 0.040 g/kg BW every 2 day	NA	Reduced TNF-α and IFN-γ levels	[199]
19	RCT	Healthy, non-smoking volunteers (*n* = 11)	3 wk	CrB juice between meals (250 mL per day) (560 mg GAE/100 mL)	NA	Increased serum antioxidant capacity with no significant change in the blood lipid profile	[200]
20	RCT	Men with the diagnosed mild hypercholesterolemia (*n* = 58)	6 wk	CB juice between meals (250 mL per day) (560 mg GAE/100 mL)	NA	Improved lipid profile with reduced lipid peroxides (LPO), C-reactive high sensitivity protein (hsCRP), homocysteine,	[201]
21	3-arm, DB, parallel RCT	Healthy male volunteers (*n* = 66)	12 wk	CB extract” capsules (containing 116 mg total (poly)phenols). CB whole fruit” capsules (containing the equivalent to 10 g of the whole CB fruit, and 12 mg of total (poly)phenols)	NA	Increased *Anaerostipes, Bifidobacterium*, *Faecalibacterium*, and *Clostridium* genera	[202]
	(I) Black Currants (BCT) (Animal studies)						
1	RCT	Old male Sprague-Dawley (SD) rats (*n* = NS)	Single meal challenge test	BCE 5 mg/kg.BW (1 mg D3R/kg.BW)	Normal diet with IP administration of glucose solution (2 g/kg)	Improved hyperglycemic and hypoinsulinemic condition	[203]
2	RCT	Male KK-Ay (*n* = 16)	7 wk	BC extracts (2 g/Kg.diet) (equivalent to delphinidine-3-glucoside (D3R) 2 g/Kg.diet)	NA	Improved glucose tolerance with increased GLP-1 concentration, and upregulation of AMPK-α and prohormone convertase 1/3(GLP-1 precursor)	[204]
3	RCT	Male C57BL/6J mice (*n* = 48)	8 wk	Diet supplemented with 1% BC powdered extract (32% anthocyanins)	HFD (60 kcal% fat diet)	Protective effect of BC anthocyanins against obesity and associated insulin resistance.	[205]
4	RCT	Male C57BL/6J mice (*n* = 24)	12 wk	HF/HC diet supplemented with 0.1% of BCE (containing 25% anthocyanins and 40% polyphenols) by weight	AIN-93M high fat/high cholesterol (HF/HC) diet (16% fat, 0.25% cholesterol by weight; 55.7%, 125.5% and 31.8% energy from carbohydrate, protein and fat, respectively; 4529 kcal/Kg	Reduced BW and adipocyte size of the epididymal fat, energy expenditure and mitochondrial biogenesis genes	[206]
5	RCT	Male New Zealand white rabbits (*n* = 20)	4 wk	Diet supplemented with 1.5% BC polyphenolic extract	HFD (10% lard) was 17% from protein, 32% from fat and 51% from carbohydrates	Reduced concentration of putrefactive metabolites, β-glucuronidase activity, ameliorated hyperlipidemia, and antioxidative capacity	[207]
6	RCT	Sprague–Dawley male rats (*n* = 40)	4 wk	2 mL of BC extract (containing 30 mg BC /kg BW) or 2 mL of CAM30 extract (containing 13.4 mg CAM30/kg body weight), respectively, three times weekly by oral gavage	NA	Reduced β-glucuronidase activity and undesirable bacteria in the caeca. Increased lactobacilli and bifidobacterial gut species	[208]
7	RCT	Male Sprague-Dawley (SD) rats (*n* = 40)	8 wk	BC extract 100–300 mg/kg.BW.day administered orally	High-fructose (HF) diet (60% fructose diet)	Improvements in hypertension, dyslipidemia, insulin resistance, and obesity	[209]
8	RCT	Male Sprague-Dawley rats (*n* = 128)	6 wk	Diets with dietary fiber and BC extracts (Currantex 30) (containing total anthocyanin 32% (*w/w*))	NA	Increased intestinal population of SCFA and total beneficial bacterial population	[210]
	(Human studies)						
9	RCT	Healthy volunteers (*n* = 30)	2 wk	BC extracts (1500 mg/day; 375 mg × 4 capsules) BC powder CAM30 (672 mg/day; 168 mg × 4 capsules)	NA	Increased intestinal population of SCFA and total beneficial bacterial population	[211]
10	DB, CO, RCT	Healthy subjects (*n* = 26)	Single meal challenge test	Apple and BC polyphenol-rich drinks (1200 mg apple polyphenols (AE), or 600 mg apple polyphenols + 600 mg BC anthocyanins (AE+BE))	Standardized high-carbohydrate meal 100 g of white bread	Reduced Postprandial insulin, C-peptide and GIP, GLUT and SGLT1-mediated glucose transport	[212]
11	DB, CO, RCT	Healthy subjects (*n* = 22)	Single meal challenge test	Low-sugar-BC drink containing 300–600 mg anthocyanins	Standardized high-carbohydrate meal 100 g of white bread	Reduced postprandial insulinemia, glycemia, and incretin secretion	[213]
12	RCT	Healthy participants (*n* = 17)	6 d	BC powder 6 g/day with water	NA	Improved postprandial AUC of glucose and insulin	[214]
13	DB, CO, RCT	Endurance-trained females (*n* = 16)	7 d	BC extract 600 mg/day	NA	Increased fat oxidation	[215]
14	RCT	Healthy sedentary male and female participants (*n* = 40)	Single meal challenge test	BC juice 200 mL/participant	standardized meal bar to consume for breakfast at least 1 h prior to starting the trial.	Supported positive affective responses	[216]
15	parallel, four-arm, study design + DB, CO parallel trial	Healthy individuals (*n* = 24) (*n* = 32)	A single meal challenge study	Two opaque gelatin capsules containing BC anthocyanin (3.2 mg/kg total anthocyanins)	NA	Dose-dependent increase in plasma anthocyanins and recovery from exercise-induced oxidative stress	[217]
(I) Maqui berries (MqB) (Animal studies)							
1	open exploratory study	Pre-diabetic volunteers (*n* = 43)	Single dose study	A single dose of Delphinol Capsules bearing either 60, 120, or 180 mg Delphinol on each day with one-week washout period	NA	Dose dependently lowered basal insulinemia and glycemia	[218]
2	RCT	Male balb/c mice (*n* = NS)	7 d	MqB extracts (25, 50 and 100 mg/kg.BW)	NA	Ameliorate the oxidative stress condition	[219]
3	RCT	Male C57BL/BJ mice	12 wk	MqB anthocyanins (ANC), Labrasol/water: 66/34 + ANC (LAB + ANC)	HFD (60% calories from fat)	Decreased glucose production, down-regulation of gluconeogenic enzyme	[220]
4	DB, CO, RCT	Fifty overweight volunteer smokers (*n* = 42)	4 wk	3 capsules of 150 mg standardized maqui berry extract containing 54 mg of anthocyanin daily (equivalent to 162 mg anthocyanins/day)	NA	Reduced levels of Ox-LDL in the anthocyanin group	[221]
5	RCT	Male C57BL/6Nhsd mice (*n* = 18)	4 wk	MqB derived Delphinidine (15 mg/kg.BW) daily	High-fat diet and high-carbohydrate drinking water (45% kcal from fat)	Reduced TG accumulation with no effect on metabolic alterations related glucose metabolism	[222]
6	Prospective observational study	Middle-aged participants (*n* = 21)	8 wk	Two tablets per day of an MCN (Eonlipid) (containing maqui, 300 mg in each tablet)	NA	Improvement of most atherogenesis and oxidative stress biomarkers	[223]
7	CO, RCT	Healthy male subjects (*n* = 11)	ONCS	Intake of 250 mL of the MqB drink containing an number of total polyphenols ~1000 µmol equivalents of gallic acid	Meals containing food-grade glucose and rice, containing 50 g of carbohydrates by each meal	Reduced glycemic indexed for high-carbohydrate diets.	[224]
8	RCT	C57BL/6J littermates’ male mice (*n* = 23)	16 wk	HFD supplemented with 4–5 mg of MqB polyphenols/ 10–15 kcal per day	HFD (45% calories from fat)	Reduced body-weight-gain, improved glucose tolerance and insulin resistance. Differential expression of genes involved in fatty acid oxidation, de novo lipogenesis, thermogenesis, and multilocular lipid droplet formation	[225]

Note: Acai berry, AB; AB juice, ABJ; ATP-binding cassette sub-family G member 8, ABCG8; AMP-activated-proteins kinase-α, AMPK-α; AB extracts, ABE; ATP-binding cassette sub-family G member 5, ABCG5; vascular cell adhesion molecule-1 VCAM-1; Apolipoprotein B, ApoB; Area under curve, AUC; Bilberry, BB; BB juice, BBJ; BB extracts, BBE; Black currant, BCT; BCT juice, BCTJ, BCT extracts, BCTE; Blueberry, BlB; BlB juice BlBJ; BlB extracts, BlBE; body weight, BW; C-reactive high sensitivity protein hsCRP; catalase CAT; Cytochrome P450 Family 7 Subfamily A Member 1, CYP7A1; Chokeberry, CB; cranberry, CrB; Cross-over, CO; cross-sectional, CS; cyanidin-3-*O*-glucoside, C3G; day, d; Double-blind, DB; Freeze-dried, F/D; High-fat-diet, HFD; low-fat-diet, LFD; glucose transporter 1, GLT; glucagon-like-peptide 1, GLP1; Glutathione peroxidase GPx; glutathione reductase GSH-x; 3-hydroxy-3-methylglutaryl-CoA, HMG-CoA; interferon alpha *IFN*-*α*; Intercellular Adhesion Molecule 1, ICAM-1; interleukin, IL; Lingonberry, LB; Low-fat-diet, LFD; Low-Density Lipoprotein (LDL) Receptor (LDL-R); Monocyte Chemoattractant Protein 1 (MCP-1); Mulberry, MB; Maqui berry, MqB; Ox-LDL, nonalcoholic fatty liver disease (NAFLD); oxidized low-density-lipoproteins; oxLDL-C; polyunsaturated fatty acids, PUFA; thiobarbituric acid reactive substances (TBARS); total glyceraldehyde, TG; total cholesterol, TC; Tumor necrosis factor, TNF-α; single-blinded, SB; Superoxide dismutase, SOD; one-time-challenge-study, OTCS; placebo-controlled, PC; Peroxisome proliferator-activated receptor-α, PPARα; phosphoenolpyruvate carboxykinase (Pepck); Raspberry, RB; Randomized controlled trial, RCT; respiratory quotient (RQ), short-chain fatty acids, SCFA; sodium glucose transporter protein, SGLT; Sterol regulatory element-binding protein, SREBP-1c; weeks, wk.

**Table 2:** A comprehensive list of potential health promoting individual anthocyanins and phenolic compounds with their quantities found in berries or berry products.

Compounds	Bilberry (mg/100 g fw)	Blueberry (mg/100 g fw)	Cranberry (mg/100 g fw)	Raspberry (mg/100 g dw)	Mulberry (mg/100 g fw)	Lingonberry (mg/g DE)	Blackberry (mg/g DE)	Strawberry (mg/100 g fw)	Goji Berry (mg/100 g dw)	Acai Berry (mg/100 g dw)	Black Chokeberry (mg/100 g dw)	Black Currant (mg/100 g fw)	Maqui Berry (mg/100 g fw)
References	[38,51]	[10,35,36,37,38,39]	[226,227,228,229]	[230,231,232]	[233,234,235]	[236,237]	[131,238,239]	[240,241]	[62,123,242,243]	[244,245,246]	[247]	[248,249]	[219,250,251]
Cyanidin	18–290	-	-	-	-	-	-	27–175	27.5	-	-	-	22.8–26.0
Delphinidin	29–280	-	-	-	-	-	-	-	-	-	-	-	105.0–120.3
Quercetin	1.5–8	0.07 *	104	-	0.3–10.04	-	-	0.09–0.54	-	39.02	37-400	-	-
Myricetin	nd-3	-	69	-	-	-	-	0.05–0.77	-	-	-	-	-
*p*-Coumaric acid	1–9	-	0.25	67.03–2792.6	0.3–4.2	0.13	-	2.64	0.07–0.22	-	-	-	-
*m*-Coumaric acid	7–30	-	-	-	0.3–14.2	-	0.93	-	-	-	-	-	-
Sinapic acid	-	-	0.211	-	-	-	-	0.61	-	-	-	-	-
Gallic acid	-	-	-	3–72.2	3.8–8.6	-	-	26.5–47.54	-	701.6	-	-	75
Ascorbic acid	-	-	0.011	2.4–5.34	-	-	-	-	-	-	-	-	-
Ferulic acid	-	-	0.087	-	5.3–294	-	-	0.95	753.6	2.46	-	-	-
Chlorogenic acid	-	3.08 *	-	177.4	4.3–22.3	-	-	0.35–1.10	-	37.65	-	-	-
Protocatechuic acid	-	-	-	-	3	-	-	-	-	-	-	-	-
5-*O*-Caffeoylquinic Acid	-	-	-	-	283–1735	-	5.57–8.88	-	8.4–37.9	-	346–413	-	-
1,3-di-*O*-Caffeoylquinic Acid	-	-	-	-	0.2–0.3	-	0.15–0.22	-	0.6–4.27	-	13–508	-	-
Caffeic acid	-	-	0.15	2.41–5.31	1.3–9.2	0.26	-	0.52	0.76–1.52	8.12	-	-	-
Protocatechuic acid	4–8	-	-	-	-	-	-	-	-	-	-	-	-
Ellagic acid	-	-	120	1151.7	23.9	-	2.012	2.72	-	-	-	-	-
Benzoic acid	-	-	4.7	-	-	3.79	-	-	-	-	-	-	-
*p*−Hydroxyphenylacetic acid	-	-	0.007	-	4.3–12.9	-	-	-	-	-	-	-	-
2,3-Dihydroxybenzoic acid	-	-	0.003	-	12.9	-	-	-	-	28.18	-	-	-
2,4-Dihydroxy benzoic acid	-	-	0.04	-	-	-	-	-	0.13–0.51	3.37	-	-	-
Vanillic acid	-	-	0.05	3–4.41	-	-	-	2.91–3.1	4–6.37	57.7	-	-	-
Trans-cinnamic acid	-	-	0.02	-	-	-	-	-	-	-	-	-	-
*O*-Hydroxycinnamic acid	-	-	0.089	-	-	-	-	-	-	-	-	-	-
*p*-Hydroxybenzoic acid	-	-	0.021	-	-	-	-	-	-	172	-	-	-
Resveratrol	1–12	-	-	-	-	0.13	-	-	-	-	-	-	-
Epigallocatechin	-	-	1.5	-	25.6	-	-	-	-	-	-	-	-
(+/−)-Catechins	6–7	-	4.5	129.3	-	-	-	19.56–135.19	106.6	49.1	593	-	-
(+/−)Epicatechin	6–7	-	4.5	791.7	0.2–24	-	-	1.07	-	44.6	6767	-	-
Gallocatechin gallate	-	-	0.4	-	10.2–63.7	-	-	-	-	-	-	-	-
Epigallocatechin gallate	-	-	1.9	-	4.5–8.4	-	-	5.65	-	-	-	-	-
Delphinidin 3-galactoside	167.1	23.4	-	-	-	Up to 1.25	-	-	-	-	-	52 ***	-
Delphinidin 3-glucoside	169.1	15.4	-	-	-		26.8–29.40	-	-	-	-	839 ***	389.9
Cyanidin 3-galactoside	122.6	4.2	2	-	-		-	-	-	-	105–2407	-	-
Delphinidin 3-arabinoside	152.3	-	-	-	-		-	-	-	-	-	-	-
Cyanidin 3-glucoside	130.4	2.6	0.1	15.02–53.94	4.72		-	-	-	998.74	5–113	327 **	679
Petunidin 3-galactoside	50	11.7	-	-	-		-	-	-	-	-	103 ***	-
Cyanidin 3-arabinoside	110.6	3.5	1.4	-	-		-	-	-	-	215–1148	-	-
Petunidin 3-glucoside	101.9	12.4	-	-	-		10.02–15.25	-	-	21	-	-	-
Peonidin 3-galactoside	13.3	1.8	2.8	-	-		-	-	-	-	-	-	-
Petunidin 3-arabinoside	23.9	9.3	-	-	-		-	-	-	-	-	-	-
Peonidin 3-glucoside	56.7	2.1	0.3	-	-		2.04–3.62	-	-	193	-	71 ***	-
Malvidin 3-galactoside	27.5	34.9	-	-	-		-	-	-	-	-	-	-
Peonidin 3-arabinoside	4.5	1	1.1	-	-		-	-	-	-	-	-	-
Malvidin 3-glucoside	67.7	31.2	-	-	-		9.49–10.57	-	-	-	-	-	-
Malvidin 3-arabinoside	12.8	34.7	-	-	-		-	-	-	-	-	-	-
Quercetin-3-galactoside	-	-	70.4	-	-		-	-	-	-	-	-	-
Quercetin-3-α-arabinopyranoside	-	-	34.4	-	-		-	-	-	-	-	-	-
Quercetin-3-rhamnoside	-	-	41.6	-	-		-	-	-	-	-	-	-
Kaempferol-3-glucoside	-	-	5.6	-	-		-	5.12–17.67	-	-	-	-	-
Myricetin 3-α-arabinofuranoside	-	-	37.5	-	-		-	-	-	-	-	-	-
Quercetin 3-*O*-glucuronide	-	-	-	717.57	-		-	9.4–39	-	-	-	-	-
Quercetin pentoside	-	-	-	252	-		-	-	-	-	-	-	-
Cyanidin-3-*O*-sophoroside	-	-	-	43.27–800.3	-		-	-	-	-	-	-	-
Cyanidin-3-*O*-rutinoside	-	-	-	5.49–104.58	2.73		-	-	-	433.98	-	1693 ***	-
Pelargonidin-3-glucoside	-	-	-	-	0.14		-	-	-	-	-	-	-
Quercetin 3-*O*-rutinoside	-	-	-	-	192–398		-	-	0.9–23.2	-	-	1.8–2.37	-
Quercetin 3-*O*-galactoside	-	-	-	-	0.2–345		-	-	-	-	-	-	-
Quercetin 3-*O*-glucoside	-	-	-	-	72.4–345.7		0.23–0.88	9.8–25.1	16.9–90.9	44–3756	-	1.5–2.0	-
Kaempferol 3-*O*-glucoside	-	-	-	-	35.5–478		-	5.96–14.39	0.5–1.94	-	-	-	-
Pelargonidin 3-*O*-rutinoside	-	-	-	-	17.8–290		-	-	-	-	-	-	-
Delphinidin-*O*-(pentosyl)hexoside	-	-	-	-	-	-	0.82–1.88	-	-	-	-	-	-
Delphinidin-*O*-rhamnoside	-	-	-	-	-	-	2.14	-	-	-	-	-	-
Malvidin-*O*-pentoside	-	-	-	-	-	-	1.08–2.13	-	-	-	-	-	-
Malvidin-*O*-rhamnoside	-	-	-	-	-	-	0.13–0.63	-	-	-	-	-	-
Caffeoylisocitrate	-	-	-	-	-	-	0.35	-	-	-	-	-	-
Caffeic acid-*O*-hexoside	-	-	-	-	-	-	0.4–0.56	-	-	-	-	-	-
Myricetin-*O*-hexoside	-	-	-	-	-	-	0.19–0.29	-	-	-	-	29	-
Pelargonidin-3-glucoside	-	-	-	-	-	-	-	17.82–20.85	-	-	-	-	-
Pelargonidin-3-malonylglucoside	-	-	-	-	-	-	-	5.51–8.16	-	-	-	-	-
Pelargonidin-3-glucoside	-	-	-	-	-	-	-	114–348	-	17.58	-	-	-
Pelargonidin-3-rutinoside	-	-	-	-	-	-	-	18–62	-	-	-	-	

Note: Atmospheric-pressure chemical ionization, APCI; Diode array detector, DAD; dry extracts, DE; dry weight, dw; Electron spray ionization, ESI; fresh weight, fw; Hexahydroxydiphenoyl, HHDP; High pressure liquid chromatography, HPLC; Liquid chromatography, LC; Lycium barbarum glycoprotein, LbGp; Lycium barbarum polysaccharides, LBP/LBPC/LBPA/LBPF; Mass spectrometry, MS; Nuclear magnetic resonance, NMR; reverse phase, RP; photodiode array detector, PDA; Quadrupole Time-of-Flight Mass Spectrometry, QTOF-MS; Ultra High pressure liquid chromatography, UPLC. * mg compound/mg extract. ** mg/100 g of sample dw. *** nmol/g.

## 4. Bilberries

Bilberries (BBs, *Vaccinium myrtillus*) are rich in quercetin, anthocyanins, tannins, catechins, vitamins, and pectins [252]. However, the most important classes of compounds considered responsible for the therapeutic role of BB/BB extracts (BBEE) are phenolic acids and anthocyanins. The majority of compounds belonging to these two classes are presented in Table 2. The phenolics of blueberries varied widely and comprised of 0.3% of fresh fruits, which usually ranged from 48 to 304 mg/100 g of fresh fruit. Among the phenolic acids, the most abundant phenolic acids were ascorbic acid, chlorogenic acids, and 3-caffeoylquinic acid followed by caffeic, ferulic, ellagic, and gallic acids. Among the free phenolic acids, chlorogenic acids and ascorbic acids are of prime importance with reference to their health promoting activities [39]. Additionally, at least 15 different BB anthocyanins have been identified including the antidiabetic anthocyanin aglycones, which constituted >70% of the total anthocyanin of BB (Table 2) [17,38,39]. BB anthocyanins showed excellent in vitro α-amylase and α-glucosidase inhibitory activities, reducing or preventing intestinal glucose absorption, and redirecting lipoprotein metabolism regulator enzymatic activities [43]. BB anthocyanins also inhibited advanced glycation end-product (AGE) formation, a severe diabetic complication. The main bioactive compounds considered responsible for inhibiting AGE activity were chlorogenic acid, quercetin-3-galactoside, quercetin-3-arabinoside, quercetin-3-glucoside, quercetin glycoside, quercetin-3-rhamnoside, myricetin glycoside 4, myricetin, and procyanidin b2 biomarkers [253]. BB polyphenols regulate hexose transport via GLUT2 and Na-glucose co-transporter 1 (SGLT-1), which assists glucose uptake. In other studies, GLUT2-mediated hexose transport was impeded by BB-derived flavones [48,254]. Cermak et al. [255] also reported that quercetin-3-*O*-glucoside and quercetin-4-*O*-glucoside decreased intestinal hexose absorption by inhibiting SGLUT1 in pig jejunum brush-border-membrane vesicles.

In one of the in vivo studies, supplementation with bilberry extract (BBE) reduced fasting blood sugars (FBS), total glyceraldehyde (TG), TC, and LDL-C levels. BB ingestion increased islet of Langerhans size and minimized retinopathy prognosis. BBE ingestion improved insulin sensitivity and hypoglycemia by upregulating AMPK, which upregulated GLUT4, PPAR-α, ACOX, and carnitine palmitoyltransferase-1 and ACPT-1A, which is synonymous to the suppression of glucose production and increased insulin sensitivity [15]. In another crossover study, the lyophilized BBE showed an 18% decrease in (incremental rise of) plasma glucose levels in overweight/obese diabetic humans, accompanied by decreased plasma insulin levels [48]. Recently, Alnajjar et al. [49] also reported that BBE anthocyanins reduced plasma glucose, oral glucose tolerance test (OGTT), TC, high-density lipoprotein cholesterol (HDL-C), LDL-C, TG, and inflammatory adipokine [leptin, TNF-α, and high-sensitivity CRP (hs-CRP)] levels, without affecting the plasma Trolox equivalent antioxidant capacity (TEAC). The anti-inflammatory role of BB was also witnessed when BB juice (BBJ) consumption in healthy adults also reduced NF-κB-regulated inflammatory mediator expression (CRP, IL-6, IL-15, and monokine induced by gamma-interferon) and increased plasma levels of quercetin (by 32–51%) and *p*-coumaric acid [51]. Later on, Kolehmainen et al. [53] examined the anti-inflammatory mechanism associated with BB consumption and reported the regulation of cytoplasmic ribosomal protein expression and the toll-like receptor (TLR) signaling and β-cell receptor signaling pathways, with decreased proinflammatory macrophage and monocyte functional gene expression including C-C chemokine receptor 2 and monocyte-to-macrophage differentiation. Kim et al. [127] also reported that daily BBE consumption reduced vascular permeability by reducing vascular endothelial growth factor levels in diabetic rats, in addition to restoring tight junction protein expression including claudin-5, zonula occludens-1, and occludin [127].

An accumulated number of evidence has also suggested that BB(E) intake is also helpful in relieving the oxidative stress and oxidative stress-related complications in obese and (pre)-diabetic subjects (Table 1). BBE administration alleviated stress-induced liver damage by decreasing plasma alanine aminotransferase (ALT), malondialdehyde (MDA), and nitric oxide (NO) levels and increasing glutathione (GSH) and vitamin C levels [45]. Capillary albumin filtration (CAF) is an early diabetic complication, associated with neuropathy and hypertension. BB anthocyanins prevented experimentally-induced-CAF, improving vision and retinopathy, and remarkable CAF reductions were observed among diabetic patients [46,48,256]. The suggested mechanism for inhibiting CAF involves BB anthocyanosides, which reduced aldose reductase activity and acted as strong antioxidants or pro-reductants, inhibiting AMP and guanosine monophosphate phosphodiesterase by scavenging superoxide anions [256]. Albumin retention (AR) was assessed by the isotopic CAF test in STZ-induced diabetic rats after anthocyanoside-rich BBE administration [46], and BBE treatment was found to reduce and maintain reduced AR (14% to 1.3%) and low-frequency/high-frequency (LF/HF) ratio values in diabetic rats, without toxic effects [47]. BB-derived phenols increased the population of beneficial SCOA-producing gut bacteria (*Lactobacillus* spp. and *Bifidobacterium* spp.) and reduced bacterial metabolic syndrome biomarker genera including Enterobacteria. The dysbiosis symbolic Firmicutes/Bacteroidetes ratio, IR, and obesity-led-dysbiosis also decreased following BB consumption [49]. BB added to a fermented oatmeal drink caused a high glucose response, with a significantly reduced insulin index (Table 1) [50].

## 5. Cranberries

Cranberries (CrBs, *Vaccinium macrocarpon*) have also been intensively investigated for their proclaimed favorable cardiometabolic and dysmetabolic syndrome effects, likely due to phytochemicals such as oligosaccharides, procyanidins, and anthocyanins. A comprehensive list of potential well-known antioxidative, antidiabetic, and anti-inflammatory compounds found in CrB (products) or CrB extracts (CrBE) used in clinical or non-clinical interventional studies are listed in Table 2 [226,227,228]. The purified fractions of procyanidins were more antidiabetic potent than the anthocyanin and oligosaccharide fractions [257]. With respect to individual compounds, quercetin-3-galactoside, 5-caffeoylquinic acid, and quercetin-3-rhamnoside were the major compounds comprising 75–77% of total flavonols of cranberry whilst 4-caffeoylquinic acid, 3-caffeoylquinic acid, quercetin-3-arabinopyranoside, myricetin3-galactoside, quercetin, quercetin-3-arabinofuranoside, and quercetin-3-benzoylgalactoside were found in the least amounts. Many authors have initially described the in vitro antidiabetic/antiglycation activities of cranberry extracts or its products [226,257]. Barrett et al. [227] isolated ellagitannins and proanthocyanidins and demonstrated their dose-dependent inhibition of α-amylase and glucoamylase activities. CrB powder from stress-adapted portions of cranberry juice (CrB-JSB) showed increased α-amylase and glucoamylase activities compared with CrB powder, and CrB-JSB (200 mg/mL) also showed anti-hypertensive properties by inhibiting the angiotensin I-converting enzyme (ACE-1) activity [228]. Podsedek et al. [258] found that CrB extracts inhibited pancreatic lipase activities more potently than other berries, but digestive enzyme inhibitory activities were less potent. Purified CrB proanthocyanidins and oligosaccharides also reduced the levels of HbAC1 levels from 7.05% to 5.75, 5.55, and 5.45% in the hemoglobin-glucose assay, whereas the recommended HbAC1 value should be below 7%, according to the American Diabetes Association. Reduced glucose-induced AGE formation during middle glycation stages was also observed during the human serum albumin (HSA)-methylglyoxal and HSA-glucose assays [257]. CrB-derived phenolic-rich extracts decreased fluorescent AGE generation by almost 60%, which was more effective than the other berry anti-AGE activities of raspberries, apples, grapes, and strawberries. The CrB anthocyanin and procyanidin fractions also decreased fluorescent AGE generation in an arginine-methylglyoxal model by 53.3 to 56.8% [226]. The CrB oligosaccharide-rich fraction showed concentration-dependent anti-glycation activity, which reduced AGE formations by 53.3 to 56.8%, respectively, almost as strongly as the reference compound [259].

The hypoglycemic, hypo-insulinemic, and hypolipidemic properties of CrB or its byproducts have also been reported in many clinical interventions (Table 1) [5,56,57,58,59,60,260]. Low-calorie dried cranberry (LCDC, 40 g) consumption after HFD reduced hyperglycemic and hyperlipidemic conditions, halted increases in IR/HOMA-IR and inflammatory biomarkers (TNF-α IL-6, IL-2, IL-10, IL-18, malondialdehyde-MDA) in adipose tissue, and lowered plasma lipid oxidation and oxidative stress biomarker levels in the treated group [56]. After testing LCDC, sweetened, dried CrBs (SWDC) consumed by non-insulinemic diabetic patients also reduced plasma glucose levels when compared with white bread (WB) and unsweetened dried CrBs (USCB) [260]. The plasma insulin peak following SWDC consumption appeared earlier than the insulin peaks for WB or USCB consumption and was significantly lower than those for WB and USCB. Bread consumption induced higher insulin and postprandial glucose responses, which could be diminished by incorporating CrBs [58,59,60,260]. CrB extracts (CrBEs) also halted visceral adiposity and weight gain in HFD-fed C57BL/6J mice, and improved HFD-induced hypercholesterolemia, hypertriglyceridemia, antioxidant defense mechanisms, and hepatic oxidative stress and normalized the NF-κB/IκB ratio [54]. Long-term CrBE consumption effects were also investigated [55,63], and the addition of CrBE to normal chow delayed age-related basal plasma insulin concentration declines [63]. CrBE supplementation also improved glucose responsiveness and increased insulin concentrations (7.6%) in rats, without significant HOMA-IR changes. CrBEs also induced duodenal homeobox 1 and insulin expression within islets, which enhanced insulin release, suggesting insulinotropic effect of cranberry intervention [55]. CrBEs showed the anti-obesity effect by inducing the LDL receptor expression, resulting in increased hepatic cholesterol uptake and promoted cholesterol binding to bile acids, causing increased fecal cholesterol excretion [57]

CrBJ consumption was also examined in randomized clinical studies (Table 1). Healthy adults who consumed CrB juice (CrBJ) also showed reduced proinflammatory CRP levels [61]. Daily CrBJ supplementation for 60 days increased paraoxonase-1 (PON-1) and apolipoprotein (Apo)A-I expression (dysfunctioning of PON-1 and apoA-I results in glycation in T2DM patients) accompanied by decreased blood glucose and ApoB levels in T2DM patients. CrBJ inhibited GLUT-4-mediated gastric glucose uptake and aldose reductase, α-amylase, and α-glucosidase activities and protected LDL-C against oxidation [60,64,228]. Moreover, both routine-calorie CrBJ (RCCJ) and high-calorie CrBJ (HCCJ) are enriched in hexoses and sugars, which could limit their use by diabetic individuals. Therefore, low-calorie CrBJ (LCCJ) was examined in glycemic and insulinemic T2DM patients by Wilson et al. [58,59] and Novotny et al. [65]. LCCJ consumption did not affect LDL-C, HDL-C, or TC levels; however, ApoA-I, ApoA-II, ApoB, and TG levels were reduced in the treated group. Individuals with higher baseline TG or HOMA-IR values experienced more pronounced drops in TG and HOMA-IR than others [65]. Serum HbA1c levels were reduced by 11.4% and 6.02% following RCCJ and RCCJ enriched with omega-3 fatty acid consumption. Omega-3 fatty acid-enriched RCCJ also increased HDL-C levels by 21.1% compared with the baseline [63]. Additionally, folic acid consumption combined with LCCJ decreased plasma homocysteine levels and increased adiponectin and folic acid levels without any change in inflammatory biomarker levels (IL-6, IL-10, IL-18, and TNF-α) [66].

In summary, CrB consumption exerted antimetabolic syndromic effects by downregulating GLUT2 and GLUT4 expression and increasing hepatic cholesterol uptake. Diet-induced weight gain and low-grade inflammation were counteracted by the prevention of TG accumulation and strengthened antioxidative defense mechanisms. The other proposed possible mechanisms of action of CrB, or its products, consumption include reduction and inhibition of ACE-I activity and oxidative stress, accompanied by improvements in endothelium-dependent vasodilation. Furthermore, CrB-derived bioactive compounds including quercetin, inhibited microsomal TG transfer protein (MTP), preventing ApoB-containing lipoprotein assembly. Quercetin also lowered proinflammatory CRP expression in a transgenic mouse model and decreased cytokine-induced CRP expression in Hep3β cells and Chang liver cells [68,69,261], which was analogous to weight loss- and polyunsaturated fatty acid (PUFA)-rich Mediterranean diet-induced CRP suppression [65,261]. Additionally, CrB consumption has beneficial effects on the gut microbiome. HFD reduced Bacteroidetes and increased Firmicutes populations in C57Bl/6J mice, which was reversed by CrBEs intake. CrBEs also increased the Akkermansia gut population, which may prevent HFD-induced increases in circulating pro-inflammatory lipopolysaccharides (LPS) [54].

## 6. Raspberries

Raspberries (RBs), especially red RBs (*Rubus idaeus* L.), are rich in fiber and potent therapeutic phytochemicals that have rendered raspberries as a functional food for metabolic syndrome [199]. The phytochemicals of raspberries provide the healthy and protective affects to its consumers by influencing the cell signaling pathways that affect transporters, receptors, cellular events, and gene expression. These health promoting RB phytochemicals belong to ellagitannins and anthocyanins (Table 2) [262]. Among these two classes, RB anthocyanins are major contributors to health promoting bioactivities. The anthocyanins of RB are cyanidin-based, but with dissimilar glycosidic units. The pelargonidin-based anthocyanins are only found in RB and strawberries with a sophoroside unit attachment unique to raspberries. Ellagitannins are hydrolyzable tannins that represent another major RB phytochemical group, which are hexahydroxydiphenoyl esters with quinic acid or glucose cores. Glucose cores can attach to galloyl groups, and further arrangements within hexahydroxydiphenoyl molecules yield the ellagic acids. Numerous in vitro studies have described that RB extracts (RBE) reduced lipid oxidation, LDL-oxidation, ROS generation, and DNA damage, associated with upregulated CAT and SOD enzymatic antioxidant activities [73]. Hypoglycemic studies revealed that RBEs inhibited α-amylase, with mixed effects on α-glucosidase, and aglycones and anthocyanin promoted GSIS from pancreatic cells [263,264]

Fresh RB extracts (RBEs) and freeze-dried RB powder have also been employed for in vivo evaluation (Table 1), in which oxidative stress was found to be relieved as decreased protein and lipid oxidation and damage was seen [74,75]. RB freeze-dried powder fed to obese and diabetic mice reduced ROS levels in erythrocytes by 0.87% when compared to the controls, indicating the ROS-neutralizing role of RB powder bioactive constituents during homeostasis. The RB intervention reduced ROS levels by increasing the glutathione peroxidase (GPx)/SOD ratio (2%) and GPx activity (2.13%) when compared to the placebo controls. Upregulated GPx activity also inhibited lipid peroxidation and protected against diabetes by delaying perturbed metabolism development [76]. RB juice (RBJ) given to hypercholesterolemic golden Syrian hamsters reduced plasma LDL-C levels and increased hepatic GSHPx and SOD activities by 30% and 25%, respectively [72]. Polyphenol-rich black RBs have also been combined with HCD foods for sustainable postprandial glycemic control, reducing plasma free fatty acid (FFA) and oxidative stress marker levels. RBs, combined with HCD, blunted postprandial insulinemia and ex vivo LDL-oxidation during the postprandial state, hindering glucose uptake (Table 1) [91]. Purified hydrolyzable RB tannin supplementation in rat gastritis models also demonstrated increased endogenous antioxidant defense system components and decreased inflammatory biomarkers and conditions. RB ellagic acid suppressed the specific immunoglobulin antibody response in cytotoxic cells without affecting other immunoglobulin parameters. Reduced lipid peroxidation, neutrophil infiltration, and iNOS overexpression were observed in ex vivo gastritis and Crohn’s disease models [85,86]. A recent study showed that RBE consumption mitigated carcinogenic acrylamide-induced liver toxicity in male Wistar rats. RB treatment increased plasma antioxidants enzyme levels and reduced acrylamide-induced hepatic ALT, aspartate aminotransferase (AST), alkaline phosphatase (ALP), lactate dehydrogenase (LDH), and gamma-glutamyltransferase (γ-GT) activities [265].

Limited human clinical trials have been performed with RBs, but the antidiabetic effects of RBEs and purified compounds have been examined in diabetic rat models (Table 1) [87,88]. Numerous anthocyanin and polyphenolic components have been hypothesized to affect starch digestion, altering postprandial glucose levels [263]. RB anthocyanin also enhanced insulin sensitivity, upregulated adiponectin expression, downregulated inflammatory cytokines, and altered AMPK phosphorylation, which is a T2DM therapeutic target [264]. A clinical trial examined RB intake with a HCD and reported no postprandial insulin and glucose response alterations [92,93]. In another study following HC-bar consumption, RB intake increased postprandial glucose levels, without changing peak glucose concentrations, and diminished postprandial insulinemia [91]. RB effects on IR and the underlying mechanisms in skeletal muscles were studied by Zhao et al. [87]. AMPK inactivation led to skin lipid accumulation and insulin sensitivity loss. This study found that AMPK-α1 is important for AMPK activation, and dietary RB powder inclusion increased insulin sensitivity by upregulating cytochrome C protein in AMPK-α1^+/+^ rats [87]. The supplementation of 5% RB with HFD improved insulin sensitivity by increasing IRS-1 phosphorylation at Tyr 612 and increasing the p-Akt/Akt ratio. RB intake also attenuated nod-like receptor pyrin containing 3 (NLRP3) inflammasome activation, which is a major contributor to metabolic syndrome. NLPR3 activation, combined with caspase 1, forms caspase 1p20 and caspase 1p10. Caspase 1p20 activation releases IL-1β and IL-18. RB consumption downregulated NLPR3, caspase 1p20, IL-1β, and IL-18 expression in HFD-fed mice [88]. Recently, Zou et al. [266] also reported that 5% RB powder supplementation with HFD suppressed TNF-α, L-6, IL-1β, and NF-κB p65 expression and increased GLUT4 expression and IRS-1 and Akt phosphorylation. RB powder also increased mitochondrial biogenesis genes (PGC-1α and Nrf1) and mitochondrial abundance markers (cytochrome c, citrate synthase, and cytochrome c oxidase subunit IV) [266].

The health-promoting effect of raspberry supplementation on the glycerophospholipids metabolism is also evident (Table 1). The addition of 10% freeze-dried RB to an isocaloric diet increased plasma HDL-C (1.5%) and insulin sensitivity and decreased abdominal fat (38%), blood TG, cholesterol, ROS (19%), and LDL-C (0.3%). Similarly, RB-derived cyanidin-3-glucoside upregulated GLUT4 expression, without affecting insulin sensitizer adiponectin [89]. Ellagic acid, which is unique to RB, increased insulin secretion and decreased FBS, HbA1c, and glycated urinary albumin levels. RB inclusion in HFD/HCD diminished impaired insulin tolerance and inflammatory cytokines. RB seed flour, combined with a HCD, downregulated the lipogenic gene expression of lipoprotein lipase (LPL), stearoyl CoA desaturase-1 (SCD-1), and diacylglycerol acyltransferases 2 (DGAT2) and gluconeogenesis promoting genes including PEPCK, G6Pase, sterol regulatory element-binding protein 1c (SREBP-1c), and carbohydrate response element-binding protein (ChREBP) (Table 1) [90]. RB ketones also prevented HFD/HCD-induced BW gains, reduced visceral and adipose tissue, reduced hepatic TG contents, and increased norepinephrine-induced lipolysis in white adipocytes, suppressing lipid accumulation by enhancing lipolysis and fatty acid oxidation [267]. RB supplementation in diabetic patients substantially lowered postprandial glucose levels, without affecting plasma insulin levels after a fatty meal challenge [94]. RB consumption also reduced TG levels [71]. Conflicting results regarding RB interventions and effects on metabolic syndrome biomarkers have been reported. Noratto et al. [76] found an insignificant difference in the weight gain between diabetic mice fed with and without RBs. Similarly, Kirakosyan et al. [77] and Norrato et al. [76] reported no RB intervention effects on LDL-C, fasting blood insulin, IκBα, and PPAR-γ levels. Contrasting results may be due to higher baseline weights of the subjects. However, Kirakosyan et al. showed that RB intake reduced glucose metabolisms and insulin signaling mRNA levels including MAP2K1, glycogen synthase (GYS1), hexokinase, IκBβ, phosphatidylinositol-4,5-bisphosphate 3-kinase, mechanistic target of rapamycin (mTOR), Chuk (involved in innate immunity), C-X-C chemokine receptor type 4 (involved in inflammation), LPL, GYS1, MAP2K1(involved in apoptosis), nicotinamide phosphoribosyltransferase, ApoE, PPAR-γ, and PPAR-α (involved in glucose and lipid dynamics) (Table 1) [77].

RB intake also increased gut *Lactobacillus*, which is a healthy gut marker, and increased beneficial gut intestinal SCFAs, which are colonic epithelial cell substrates and improve gut health [78]. RB consumption increased SCFA-producing bacterial populations including *Bacteroides*, *Butyricimonas*, *Ruminococcus, Akkermansia, Clostridium butyricum, Mucispirillum, Oscillibacter, Ruminococcaceae,* and *Lachnospiraceae,* which improved metabolic syndromic conditions during metformin T2DM treatment [268]. Furthermore, RB consumption time- and dose-dependently increased the gut microbial population of *Anaerostipes*, *Ruminococcus*, *Akkermansia*, *Coprobacillus*, *Allobaculum*, *Anaerovorax*, *Dorea*, *Asaccharobacter*, *Anaerotruncus*, *Coprobacillus*, *Desulfovibrio*, *Victivallis*, and *Mucispirilum,* and decreased the microbial population of *Acetivibrio*, *Anaerotruncus*, *Bifidobacterium*, *Lactococcus*, *Prabacteroides*, *Streptococcus*, *Turicibacter*, and *Acetivibrio*. Increased beneficial microbial communities as above-mentioned can reduce inflammation, obesity, metabolic syndrome, and dysbiosis [79]. Su et al. [80] reported that RB-derived pelargonidin-3-*O*-glucoside increased the gut population of *Prevotella* and improved the Bacteroidetes/Firmicutes ratio. Another more recent report concluded that there was a favorable higher population of *Akkermansia muciniphila* and Bacteroidetes/Firmicutes ratios in pathogenic free mice fed on black RB powder [81]. Conclusively, RB consumption showed antidiabetic effects, inhibiting glucosidase and amylase activities, strengthening the endogenous antioxidant defense system, reducing inflammatory biomarkers, activating AMPK, GLUT2/GLUT4, IRS-1 phosphorylation, downregulating lipogenesis and gluconeogenesis genes, and increasing epithelial mucus barrier protecting and SCOA-producing bacterial populations (Table 1) [83,84].

## 7. Mulberries

Mulberries (MBs, *Morus alba*/*Morus rubra*) are rich in cyanidin-3-glucoside, cyanidin-3-rutinoside, and pelargonidin-3-glucoside, and other anthocyanins comprising 78% of the MB polyphenolic compounds (Table 2) [269]. These purified anthocyanins from MB showed excellent glucose-lowering properties in HepG2 cells, increasing PPAR-α and AMPK phosphorylation (activation) and the p-mTOR/mTOR ratio (synonymous with the activation of insulin receptors and insulin-like growth factor 1 receptors). During metabolic syndrome, IRS-1 inactivation increases the p-p38/p38 ratio (subfamily of MAPK, which requires inflammatory cytokines for activation) and reduces PGC-1α expression (a regulator of energy homeostasis and mitochondrial biogenesis), which were abolished or reversed with MB anthocyanins treatment [269]. In addition to anthocyanins, polyphenol-rich MB methanolic extracts also showed excellent α-glucosidase inhibitory activities due to quercetin 3-*O*-rutinoside, chlorogenic acid, and cyanidin 3-*O*-glucoside [235]. Cyanidin glycosides in MBs also reportedly possess potent anti- α-glucosidase activity, which inhibit the enzyme by affecting α-glucosidase α-helix contents via cyanidin-3-glucoside (C3G) and cyanidin-3-rutinoside (C3R) domain matching [270]. HepG2 cells treated with the five most abundant MB polyphenols including C3G, 1-deoxynojirimycin, resveratrol, C3R, and oxyresveratrol showed improved glucose consumption and postprandial glucose disposal through increased glucokinase activity [271]. Another study found that 1,5-dicaffeoylquinic and dihydroquercetin acid protected cells against glucotoxicity [29]. MB extracts (MBEs) upregulated PGC-1α (38%) and FOXO1 (40%) (regulator of PEPCK and G6Pase enzymes) and downregulated PEPCK (79%) and G6Pase (37%) expression in IR model cells. MBEs also upregulated AKT2 (crucial for IRS activation and hence increasing insulin sensitivity) and glycogen synthase kinase (GSK)3β levels, with significantly increased p-AKT/AKT ratios (hence reduced IR) and increased GSK3β phosphorylation and glycogen synthase 2 (GSY2) activation [272].

In in vivo studies, MB polyphenols and polysaccharides reduced ROS levels and enhanced reductant enzymatic activities including GPx, SOD, and CAT while reducing IL-8, TNF-α, COX-2, and IL-6 release in STZ-induced diabetic mice (Table 1) [273]. MB anthocyanins also attenuated HFD-induced decreased hepatic SOD and GPx activities [95]. Yan et al. [269] reported that MB anthocyanins alleviated hypoglycemia by inhibiting ROS generation, promoting AMPK phosphorylation, activating tuberous sclerosis 2, (reducing the mTOR and ACC signaling), reducing p38-MAPK and PGC-1α expression, and increasing mitochondria and matrix metalloprotease (MMP) abundance in diabetic mice (Table 1) [97]. MB wine consumption by diabetic mice also reversed glycemic status, with reduced oxidative stress markers, proteinuria, non-esterified fatty acid contents, and lipid peroxidation and improved antioxidant defense systems [97]. MB-derived and purified cyanidin-3-*O*-β-D-glucopyranoside intervention also circumvented diabetic cytopathy by reducing oxidative stress markers of DNA modification including 8-hydroxy-2-deoxyguanosine and increasing the axonal transport of nerve growth factor [98].

The oral MBE supplementation also improved insulin signaling by decreased GSK3β, and increased GSY2, AKT, increasing p-AKT/AKT ratios in skeletal, hepatic, and adipocytes tissues of diabetic mice [272]. Oral MB fruit intake in diabetic mice also improved insulin sensitivity by upregulating (up to 3%) the IRS-1, p-IRS01/IRS-1, p-AMPK/AMPK, CCAAT-enhancer-binding proteins (C/EBP), sterol regulatory element-binding protein 1 (SREBP-1c), and PGC-1α [269,274]. Ren et al. [99] further reported that MB consumption normalized glucose metabolism by abolishing protein-tyrosine phosphatase 1B expression and activating the phosphoinositide-3-kinase (PI3K)/AKT pathway. MB anthocyanin-induced p38-AMPK-PGC-1α pathway upregulation increased thermogenesis gene activity. Anthocyanin components also downregulated lipogenesis genes including hydroxymethylglutaryl coenzyme A reductase (HMG-CA-R), SREBP-1c, and FAS [100] and activated scavenger receptor class B type 1 and ATP-binding cassette transporter (ABCA1), which transfer cholesterol.

MBEs combined with HFD demonstrated excellent anti-obesity and hypolipidemic properties. MBE supplementation reduced BW gains by 41.3% in HFD-fed diabetic male C57BL/6 mice. Serum TG, TC, HDL-C, and LDL-C levels in HFD + MBE-fed mice were lower than those in HFD-fed diabetic mice, but higher than the MBE-fed controls. Liver injury parameters (ALT and AST) were reduced in HFD + MBE-fed mice, with reduced adipose and hepatic liver lipid droplet sizes [101]. MB fruit consumption lowered TG, TC, LDL-C, and FFA levels in other studies (Table 1) [102,103]. MB-derived anthocyanin consumption decreased serum levels of inflammatory markers (IL-6, IL-1α, iNOS, TNF-α, IFN-γ, and NF-κB), thiobarbituric-acid-reactive substances (TBARS) (a lipid oxidation marker), hyperlipidemic markers (TC, glucose, TG, and leptin), insulin, and hepatic AST, ALP, and ALT levels, downregulated FAS, and increased heme oxygenase-1 (HO-1) (a cytoprotective enzyme) and antioxidant enzyme levels in HFD-fed male C57BL/6 mice (Table 1) [95,104]. Aqueous MBEs employed the hypolipidemic and hypoglycemic effects by activating the AMPK, increasing the p-AMPK/AMPK ratio (hence improving mitochondrial biogenesis), and downregulated FAS, acetyl coenzyme A carboxylase (ACC), glycerol-3-phosphate acyltransferase (GPAT), and SREBP-1 [104]. MBEs in HFD-fed male Sprague-Dawley rats prevented non-alcoholic fatty liver disease (NAFLD) by downregulating lipid/cholesterol homeostasis-related genes (FAS, ACC, GPAT, and SREBP-1) and suppressing the lipid oxidation biomarkers MDA and 4-hydroxynonenal [105,106]. Hu et al. [275] demonstrated that MBE increased nuclear factor erythroid-2-related factor 2 (Nrf2) phosphorylation and nuclear translocation, activating the Nrf2/antioxidant response element signaling pathway, which increased quinone oxidoreductase 1, HO-1, and NAD(P)H expression and promoted antioxidant enzymatic activities, thus protecting hepatocytes against palmitic acid-induced lipo-toxicity and oxidative stress.

Gut microbiota regulates dietary energy harvesting, glucose homeostasis, and lipid metabolism, especially in brown adipose tissues (BAdT). Mitochondria-rich BAdT activation can increase energy expenditure following MB-induced UCP1 upregulation and oxidative phosphorylation downregulation, releasing energy as heat. MB powder consumption reversed HFD-induced gut microbiome changes, increasing the Bacteroidetes/Firmicutes ratio and Bacteroidetes populations (*Porphyromonadaceae, Parabacteroide, S24-7, Prevotellaceae*, *Alloprevotella*, *Rikenellaceae*, *Alistipes*, *Rikenella*) and decreasing the *Proteobacteria* (*Alphaproteobacteria, Brevundimonas, Devosia, Rhodobacteraceae*, *Polymorphobacter*, *Deltaproteobacteria*, *Desulfovibrio*, *Arenimonas*), and Firmicutes (*Clostridia, Lachnospiraceae, Eubacterium, Coprococcus, Ruminococcaceae*, *Oscillibacter*, *Ruminiclostridium*) populations [107,108]. At the genus-level, MB fruit supplementation promoted SCOA/SCFA-producing and IMBD-restoration-supportive genera *Lactobacillus, Bacteroidales, Bacteroides, Allobaculum,* and *Akkermansia* growth, and suppressed *Corynebacterium, Staphylococcus, Aerococcus, Jeotgalicoccus, Facklamia,* and *Enterococcus* growth. *Allobaculum* and *Lactobacillus* protect against metabolic syndrome, and both genera increased in diabetic rats after MB intake [108]. Approximately 60 metabolites were identified in MB including flavonols, phenolic acids, flavonoids, lignans, and organic acids (Table 2) [234]. In short, MB fruit consumption upregulated/activated glucose-consumption-related pathways and insulin-sensitivity-related pathways (p-AKT/AKT ratio, glucokinase, PGC-1α, FOXO1, IRS-1, p-IRS-1/IRS-1, p-AMPK/AMPK, C/EBP, and Bacteroidetes/Firmicutes ratio) and downregulated lipogenesis-related pathways (FAS, ACC, GPAT, and SREBP-1) in skeletal, hepatic, and adipocyte tissues.

## 8. Lingonberries

Lingonberry (LB, *Vaccinium vitis-idaea*) alleviates metabolic syndrome including frequent urination and fatigue. In in vitro studies, LB extracts (LBEs) increased glucose uptake in C2C12 skeletal muscle cells by modulating AMPK activity [276]. LB polysaccharides inhibited α-glucosidase activity (by 118–136%) more strongly than the referenced acarbose [277]. In in vitro digestibility assays, LB polyphenols (7% *w*/*v*) were added to white rice, which significantly reduced glucose release [278]. Ethanolic LBEs demonstrated antiglycation activity, with AGE inhibition majorly mediated by LB cyanidin-3-galactoside, quercetin-3-galactoside, and (+)-catechin [279]. In J774 macrophages, LBEs significantly inhibited LPS-modulated NO production, without substantial effects on COX-2 or iNOS expression. Proinflammatory cytokine (IL-6, IL-1β, and TNF-α) expression was reduced by TNF-α downregulation, IκB receptor degradation inhibition, and reduced extracellular signal-related kinase 1/2 phosphorylation [280]. However, in RAW 264.7 macrophages and activated 3T3-L1 adipocytes, LBEs mitigated oxidative stress by suppressing COX-2, iNOS, TNF-αα, IL-6, MCP-1, and IL-1β expression [281].

In in vivo studies, LB consumption also improved hyperinsulinemic, hyperglycemic, and dyslipidemic conditions (Table 1) [111]. LBE consumption reduced blood glucose levels (17–25%), obesity-induced hepatic steatosis (50–60%), and plasma TG, TC, and LDL-C levels (12–18%) associated with increased GLUT4 expression and AMPK and Akt phosphorylation, increasing glucose metabolism and hepatic fatty acid oxidation [111]. LB juice (LBJ) improved low-grade inflammation and endothelial function by increasing NO availability, which is necessary for the inhibition of adhesion molecules, MCP-1, ACE-1, COX-2, and other pro-inflammatory markers [112]. The LB-rich Okinawan-based Nordic diet improved anthropometric (BW, body mass index (BMI), and waist circumference) and metabolic (HOMA-IR, IR, FBS, TG, CRP, TC, and HDL-C) parameters [119]. Linderborg et al. [120] demonstrated that LB powder consumption compensated for additional glucose and lipid consumption. LBJ intake prevented HFD-induced BW gains in C57BL/6JBomTac mice. LB supplementation reduced FBS, fasting insulin, and HOMA-IR levels (Table 1) [113,114]. Hepatic lipid accumulation and liver function parameters (ALT, TG, and cholesterol) decreased after LB supplementation, more strongly than other berries [113,114]. In a recent hyperlipidic and hypercaloric meals challenge study, the LB supplementation halted increased cholesterolemia and decreased the glycemic response, CRP, and postprandial endotoxemia [121]. In an atherosclerosis ApoE^−/−^ mouse model, whole LB consumption upregulated bile acid synthesis gene Cyp7a1, increased the cecal propionic-acid-producing bacteria proportions, and decreased triglyceridemia and atherosclerosis [115]. The insulinemic and glycemic response following oat bread consumption was also checked. The LB polysaccharide and fiber consumption, following bread consumption, reduced glucose and CRP responses [122]. Whole LB and LB nectar intake reduced postprandial glucose and insulin levels after 35 g sucrose intake, and insulin levels increased more rapidly following LB than after glucose intake. Postprandial glucose levels were also reduced following LBJ consumption. Insulin and FFA changes after LBJ consumption were similar to those observed after whole fruit consumption (Table 1) [113,114,123].

Urinary metabolomics revealed that a LBJ-containing diet increased 4-hydroxyhippuric acid and hippuric acid excretion, whereas 4-deoxythreonic acid, 3-hydroxybutanoic acid, dimethylamine, creatinine, and citric acid excretion reduced, likely due to high polyphenolic compound and benzoic acid contents in LBJ (Table 2) [124,282]. Plasma lipidomics data showed that LB consumption increased health-promoting lyso-phosphatidylethanolamines, (LPE) (16:0), lysophosphatidylcholine (LPC) (20:5), (16:1), and (22:5), and phosphatidylcholines (PC) (33:2), (32:2), (35:6), (34:4), (36:6), and (36:5), whereas obesity and diabetes symbolic sphingomyelins (SM) (34:1), (33:1), (40:3), and (38:2) were reduced. Quinate levels also increased, and plasma alanine and glucose levels decreased significantly [116]. LBE and powder supplementation of HFD downregulated the expression levels of macrophage marker endothelial growth factor-like module containing mucin-like, hormone receptor-like 1 (EMR1), and LPS-sensing TLR4 (member of the toll-like receptor family activation of which results in signaling the NF-κB pathway and inflammatory cytokine production) and upregulated tight junction-associated occluding (an integral membrane protein whose modulation is associated with cellular proliferation, differentiation, signal transduction, and migration) and proglucagon (a precursor of glucagon from α-pancreatic cells). The HFD-fed control microbiome showed the upregulation of the ATP-binding cassette (ABC) transporter, cell motility, membrane transporter, bacterial chemotaxis, bacterial motility, the two-component system, flagellar assembly, transcription, and signal transduction genes, compared with the LB-treated group [283]. LB consumption enriched genes associated with lipid metabolism, nutrient transport, energy, nucleotides, and amino acids (Table 1) [113,114,117]. At the phyla level, LB supplementation affected the diversity and population of *Firmicutes*, *Bacteroidetes*, *Proteobacteria*, and *Verrucomicrobia*. The relative abundance of *Bacteroidetes* increased, and the relative abundance of *Firmicutes* decreased significantly, reducing the obesity and diabetes symbolic *Firmicutes/Bacteroidetes* ratio [113,114,117]. At the genus level, HFD increased *Firmicutes* genera populations including *Lachnospiraceae, Oscillospira,* and *Ruminococcus*. The abundance of *Bacteroidetes* increased following LB supplementation, due to unknown members of the S24-7 family. LB supplementation increased *Parabacteriodes*, *Odoribacter*, and *Akkermansia* populations. The principal component analysis confirmed LB extract-induced gut microbial profile variations. HFD increased the population density of the genera *Oscillospira* and *Ruminococcus* and the *Lachnospiraceae* family, microbes associated with diabetes pathogenesis progression [284], which was prevented by LB fruit/powder/extract consumption [285]. *Akkermansia* population increases were associated with the abundance of *Akkermansia muciniphila* species, which are known beneficial gut microbacteria that counteract HFD-induced adipose tissue inflammation, endotoxemia, BW gain, and IR in C57BL/6 mice [286]. Liquid chromatography (LC)-tandem mass spectrometry (MS/MS)-based LB fingerprinting identified several bioactive compounds responsible for antioxidative, antidiabetic, and anti-inflammatory properties. These bioactive compounds primarily belong to anthocyanidins, flavonols, glycosides, catechins, and different conjugates of ferulic and caffeoyl acid (Table 2). Depending on aglycon weight, cyaniding-containing compounds were the major bioactive compounds followed by proanthocyanidins, which represent phenolic compounds in LB [236,237].

## 9. Blackberries

Blackberries (*Rubus grandifolius* L and *Rubus fruticosus* L.) are consumed fresh or as juices, jams, and liquors. Blackberries are enriched in health-promoting compounds (Table 2) belonging to flavanals, flavanones, flavonols (kaempferol and quercetin glycosides), anthocyanins, hydroxycinnamic acids, and caffeic acid conjugates. The high-performance liquid chromatography (HPLC)-electrospray ionization (ESI)-mass spectrometry (MS)-based *Rubus grandifolius* L. metabolic profiling revealed 50 phytochemicals including anthocyanins, hydroxycinnamic acids, flavonols, flavanones, and ellagitannins (Table 2) [131,238,239]. These blackberry-derived compounds offered an antidiabetic and anti-obesity role by inhibiting digestive enzymes (α- and β-glucosidase, aldose reductase, lipase, and α-amylase) and exhibiting anti-glycation abilities. The blackberry α-glucosidase and α-amylase inhibitory activity was superior to the reference compounds, Acarbose and 1-Deoxynojirimycin (1-DNJ) [287]. Anthocyanins are considered to be the primary mediator of blackberry extract anti-digestive activities, and glycosides are the primary inhibitors of α-glucosidase activity. The interaction between glycosides and enzymes is considered to be competitive, suggesting that glycosides bind to enzymatic active sites [288]. Cytidine glycosides from leaf and fruit *R. grandifolius* extracts reduced aldose reductase activity, which is responsible for AGE accumulation in diabetic patients via dicarbonyl activity [289]. The recorded anti-glycation activity of BB fruit extracts was IC_50_ = 1.87 mg/mL, and ellagitannins and flavonols were the most prominent anti-glycation agents [131,238,239]. HepG2 cells incubated with gut microbial-fermented blackberry metabolites (GMBB) and gastrointestinal-digested BB slurry (GIDBB) showed improved glucose uptake. Increased HepG2 uptake also increased glycogen synthesis. GIDBB and GMBB also maintained the desired cellular redox status by neutralizing ROS and restoring the mitochondrial membrane potential. GIDBB and GMBB supplementation restored glutathione levels, strengthening the oxidative defense system [290].

In in vivo studies, blackberry-derived purified anthocyanin-enriched and ellagitannin-enriched fractions decreased lipid peroxidation markers (TBARS and MDA) and increased hepatic and brain antioxidant enzyme activities (CAT, GSH, SOD, and GPx) [125]. Similarly, blackberry extract consumption attenuated the HFD-induced effects in an obesity-prone mouse model and prevented the increase in metabolic and lipidemic parameters, while reinforcing endogenous and exogenous antioxidant enzyme systems (Table 1) [126]. LPL activity, plasma glucose, insulin, and acyl-carnitines were also upregulated after blackberry consumption. Antioxidative enzyme system reinforcement correlated with the anti-inflammatory and anti-dyslipidemia potential of blackberry extracts [127]. The glycemic and lipidemic-controlling mechanisms of blackberry extracts were mediated through the downregulation of lipogenesis factors (FAS, SCD-1, microsomal triglyceride transfer protein, diglycerides acyltransferase, and adipose triglyceride lipase), energy coupling/uncoupling proteins (UCP-1, UCP-2, and UCP-3), pro-inflammatory cytokines (PPAR-α, Nrf2, IL-6, and TNF-α), and fatty acid β-oxidation genes (CPT-1a and ACOX-1) (Table 1) [127], which were maintained by long-term and chronic blackberry extract consumption. Additionally, the increases in total monounsaturated fatty acid contents of adipocytes, plasma brain-derived neurotrophic factor levels, and pro-inflammatory leptin levels in HFD-fed controls were counteracted by blackberry extract consumption [128]. Human clinical trials were also run, in which healthy human subjects were given BB fruits in addition to HFD, resulting in reduced fat accumulation and increased fat oxidation. Blackberry consumption lowered postprandial glucose and lipid levels by activating AMPK and BAdTs. [291]. Pulpy blackberry juice consumption by dyslipidemic patients decreased ApoB and hs-CRP, increased ApoA-1 and HDL-C, and left other lipid parameters unaffected [134]. In healthy subjects, blackberry juice increased exogenous and endogenous antioxidant enzymes. Cyanidin, ascorbate, total ellagic acid, urate, and R-tocopherol contributed to increased plasma and urine antioxidant capacities [125,133]. Daily blackberry consumption reduced dyslipidemia and insulinemic parameters in diabetic and obese adults [132]. Blackberry polyphenolic compounds inhibit digestive enzyme activities, physically interacting with hexose absorption transporters and modulating transporter expression at the genomic level [292]. Blackberry compounds may also modulate peripheral glucose use, damaged pancreatic cell regeneration, and enhance blood glucose withdrawal by increasing insulin sensitivity (Table 1) [109,129].

Blackberry juice was also examined in STZ-induced-diabetic male Sprague-Dawley and hamster rats (Table 1) [129]. Blackberry juice significantly reduced food and water intake, reducing the BWs of both control and diabetic rats [129]. Blackberry nectar supplementation of a cholesterolemic diet reduced hyperlipidemic parameters and hepatic lipid peroxidation [181]. Blackberry juice consumption effectively reduced triacylglycerols (−43.5%), glucose (−48.6%), and cholesterol (−28.6%) levels without side effects. Blackberry juice consumption limited lipid peroxidation in the plasma (−7.5%) and kidneys (−19.5%). Similarly, alcohol-free fermented blackberry juice (AFBBJ) was used to supplement HFD in obese C57BL/6J mice [270], which significantly reduced fat-mass gain and FBS and decreased plasma TG, TC, LDL-C, and HOMA-IR levels, while increasing β-cell function (HOMA-β) [22]. Liver function tests revealed no change in ALT, but AST increased in AFBBJ-treated mice. Genomic sequencing approaches revealed pancreatic gene upregulation, responsible for amino acid and glucose metabolism and insulin secretion regulation [22].

The intestinal bioavailability of blackberry polyphenols and resulting impact on gut microflora have also been recently investigated. The low-absorption and cecal accumulation of BB polyphenols were the main reasons for positive health effects. The cecal microbial fermentation of blackberry polyphenols generates antidiabetic and antioxidative blackberry metabolites including C3G, 2,4,6-trihydroxybenzoic acid, coumarin, and caffeic acid. The increased cecal glycoside concentration and secondary metabolites improved glucose consumption (Table 1) [290]. The increased cecal SCFA concentration suggested an increase in SCFA-producing bacteria; however, the relative abundance of different bacterial groups was not reported [130]. Blackberry treatment altered the gut microfloral composition by increasing cecal Bacteriodetes over Firmicutes. *Lactobacillus johnsonii* was abundant in both blackberry-treated and control groups, whereas *Lachnospiraceae* dominated the blackberry group, promoting glycoside metabolism. However, *Clostridiales*, *Enterococcus faecalis*, and *Bifidobacterium pseudolongum* were more dominant in the control groups [131].

## 10. Strawberries

Strawberry (*Fragaria × ananassa*) consumption has been associated with decreased risk and occurrence of metabolic syndrome, cancer, diabetes, chronic inflammation, and hypertension. The credit of these health-promoting activities goes to its rich phytochemical contents (Table 2). Many studies analytically analyzed the crude and fractionated phytochemical contents of strawberry and found strawberry rich in antioxidative, anti-obesity, antiglycation, anti-inflammatory, and antidiabetic compounds from flavanols, flavonols, anthocyanins, hydroxycinnamic acid derivatives, hydroxybenzoic acid derivatives, ellagic acid and ellagic acid glycosides, and ellagitannins (Table 2). The most surplus glucose-lowering acid moieties were malonic and *p*-coumaric acid and the most identified flavonols of strawberry were derivatives of kaempferol and quercetin glycosides. The red-coloration-granting and anti-oxidative anthocyanins of strawberries were mostly the derivatives of pelargonidin and cyanidin [240]. The hydrolysis of ellagitannins gave rise to the most important antidiabetic phytochemical called ellagic acid, which comprised more than 50% of the total polyphemolic components of strawberry. The level of ellagic acid is about 3–10 times higher in the strawberry than other berries, fruits, and nuts. It is one of the constituents due to which strawberry can regarded as a functional food [293]. In in vitro studies, strawberry ethanolic extracts inhibited pancreatic lipase activity more strongly than reference orlistat. Aqueous and ethanolic strawberry extracts inhibited adipocyte cell division and inhibited inflammatory mediator (β-hexosaminidase and histamine) release by 61.8 to 80% [294]. Strawberry polyphenolic compounds interact with glucose transporters such as SGLTI and GLUT2 and attenuate glucose uptake due to polyphenol compound competition for transporter active sites [295]. HPLC-diode array detector (DAD)-MS analysis and statistical correlations showed the contribution of pelargonidin-3-*O*-glucoside to glucose uptake inhibition. Strawberry extracts effectively inhibited uptake and transport of glucose up to 5% in HepG2 cultures [295]. Da Silva Pinto et al. [296] showed that the strawberry extract α-glucosidase inhibitory activity was superior to the α-amylase inhibitory activity. Strawberry-derived ellagitannin consumption (>50 mg/mL) sufficiently inhibited ACE activity [296]. Methanolic strawberry extracts activated p-AMPK/AMPK expression in HepG2 cells, resulting in fatty acid and cholesterol regulatory gene inactivation and phosphorylation including HMG-CoA-R and ACC. Activated p-AMPK/AMPK expression increased LDL receptor expression including PGC-1α and sirtuin 1 (a NAD^+^-dependent deacetylase that inhibit hepatic lipogenesis, stimulating FA β-oxidation, and maintaining cholesterol and bile acid levels) in HepG2 cells [297].

Numerous in vivo studies have also cited the health promoting activities of strawberry or its byproducts in animal models and human clinical trials. The intake of aqueous, alcoholic, and hydro-alcoholic strawberry extracts improved the serum glucose level, liver function (decreased serum glutamic pyruvic transaminase, serum glutamic oxaloacetic transaminase, alkaline phosphatase), lipid profile (decreased LDL-C, LDL-C/HDL-C, and LDL-C/TC ratio), and lipid oxidation markers (decreased MDA and CAT) [136,137]. Genes associated with glucose, cholesterol, and lipid metabolism [FAS, ACC, CPT-1A, malonyl-CoA, acyltransferase, ACC-α (ACACA), and acyl-CoA synthetase long-chain family member 1] were also downregulated by strawberry treatment [135]. Paquette et al. [143] used the hyperinsulinemic-euglycemic clamp methodology to examine improved insulin sensitivity and secretion after strawberry extract consumption, but did not detect improvements in fasting insulin and glucose concentrations. In animal studies, HFD supplementation with strawberry prevented weight gain without influencing food and water intake. Strawberry beverage consumption protected against postprandial lipemia by reducing TG (14%), TC (5%), and LDL-C levels (5%) in hyperlipidemic patients following HFD [144]. Sugar-rich strawberry jam consumption also attenuated glycemic index and postprandial glucose level increases in diabetic human subjects [145,298]. Strawberry jam consumption showed favorable lipid and sugar metabolism results, even compared with low-sugar strawberry jam [146]. Strawberry consumption with HCD also controlled postprandial glucose levels, affected glucose and insulin responses, and GLP-1 expression. Regular strawberry beverage and juice consumption decreased blood pressure, TC, and the TC/HDL-C ratio in diabetic patients. T2DM and CVD risk factors were also ameliorated (Table 1) [66,114,123,147]. Strawberry extracts reduced IL-6 and plasminogen activator inhibitor 1 (PAI-1) (a risk factor for atherosclerosis) levels in obese individuals after HFD/HCD, without influencing TNF-α, CRP, platelet aggregation, or fasting insulin and glucose levels [148]. In another similar study, the postprandial insulin level and inflammatory response (hs-CRP and IL-6) were reduced with increased plasma pelargonidin sulfate and pelargonidin-3-*O*-glucosidein levels after strawberry powder consumption with high-carbohydrate, moderate-fat meals [149]. In another recent study, strawberry-blueberry powder, consumed with a HFD/HCD, reduced BW gains (12.7%), visceral fat mass (18%), retroperitoneal and subcutaneous white adipose tissues (up to 10.45–16.5%), postprandial insulin and glucose levels, IR, and inflammatory markers (MCP-1, TNF-α, IL6, CRP, and PPAR-α), in male Wistar rats and C57BBL/6J mice (Table 1) [19,138]. Strawberry-blueberry powder exerted anti-adipogenic effects by regulating lipid metabolizing genes including PPAR-α and C/EBPα. Inflammatory and lipogenesis-related gene expression were reduced including TNF-α, IL6, and C/EBPα, adipogenesis-driver transcription factors (PPAR-γ), adiponectin, adipocyte fatty acid-binding protein, SREBF1, leptin, SCD-1, and FAS [138]. In another dose-response checking study, the intake of strawberry against the Western-type-meal reduced the oxidized low-density lipoproteins and post-meal insulin demand in insulin resistant patients [150].

Oxidative stress is a leading cause of metabolic syndrome and diabetes. Strawberry powder supplementation in an isoenergetic diet containing the oxidative-inducing antibiotic drug doxorubicin reversed doxorubicin-induced decreases in the antioxidants retinol and α-tocopherol and upregulated liver antioxidant enzymes including GPx, CAT, GSH, SOD, and GST (Table 1). Plasma hepatic stress biomarker levels including protein carbonyls and hydroperoxide were reduced by strawberry intake [139,152]. Strawberry-based foods containing carbohydrate, fat, and lipids increased total antioxidant levels (1.26 to 1.45 mmol/l) of the subjects while decreasing HbA1C (from 7.00 to 6.72%) levels. The plasma hs-CRP and MDA levels also decreased from 3.36 to 2.76 nmol/mL and 3.36 to 2.76 nmol/mL, respectively [153]. Strawberry powder intake prevented HFD- and stress-induced decreases in γ-aminobutyric acid levels and reduced oxidative stress and lipid oxidation markers, in male Wistar rats [140]. Fresh strawberry consumption reduced linseed oil-induced DNA damage and plasma oxidative marker levels and increased the plasma antioxidant status of pigs [299].

Strawberry intake effects on gut microbial ecology in diabetic subjects increased phylogenetic species richness (α-diversity) and global microbial composition (β-diversity) variations at the genus and operational taxonomic unit levels. *Proteobacteria*, *Actinobacteria*, and *Verrucomicrobia* were significantly altered after the strawberry intervention. Strawberry intake significantly increased the abundance of beneficial *Bacteroides* and *Actinobacteria* and decreased *Akkermansia*, *Verrucomicrobia*, *Dehalobacterium,* and *Dorea* (Firmicutes). At the genus level, the abundance of SCOA-producing *Lactobacillus* and “prebiotic-effect-giving” *Bifidobacterium* increased, whereas *Dehalobacterium*, *Dorea*, SMB53, and *Turicibacter* remained unaltered [141]. Additionally, a specific relationship between ingested flavonoids and microbial community patterns was identified [151]. Dietary flavanol and flavanone intake were positively associated with *Eggerthela lenta*. Flavonols and flavanol monomer intake was positively associated with *Adlercreutzia equolifaciens* (involved in phytochemical degradation) and inversely associated with *Flavonifractor plauti* (Gram-negative poorly understood) populations [151]. Whole strawberry powder intake increased the α-diversity of colonic inflammatory CD-1 mice, increasing *Bifidobacterium* and *Lactobacillus* and reducing pro-inflammatory *Akkermansia*, *Dorea*, and *Bilophila* [142]. The polyphenolic compounds that affected gut microbiota compositions in strawberry fruit extracts were flavanols, flavonols anthocyanins, hydroxycinnamic acid derivatives, hydroxybenzoic acid derivatives, ellagic acid, ellagic acid glycosides, and ellagitannins (Table 2) [240,241].

## 11. Goji Berries

Goji berry (GB, *Lycium Barbarum)* is a functional food and alternative therapeutic tool for T2DM treatment [155]. The major GB therapeutic phytochemicals include polysaccharides (5–8%), carotenoids (0.03–0.5%), and phenolic compounds (traces). The compounds belonging to these classes have been listed in Table 2 [123,242]. The GB is considered the best source of dipalmitin zeaxanthin carotenoids. These carotenoids showed effective protection against diabetic-induced-retinopathy [300]. The in vitro hypoglycemic tests showed the inhibitory capability of GB carotenoids was 9.6 to 82.6% and 5.7 to 15.3% for α-glucosidase and α-amylase enzymes, respectively [242]. In GB polyphenolic compounds, phenolic acids (24.7%) and flavonoids (75.3%) are major phytochemical classes. The major therapeutic flavonoids in GB are squercetin-3-*O*-rutinoside (from 7.1 to 232.7 mg/kg) and quercetin-3-*O*-hexoside (from 169.1 to 1107.7 mg/kg) whereas phenolic compounds include caffeoylquinic acid (0.34 μg/g), caffeic acid (3.73 μg/g), *p*-coumaric acid (6.06 μg/g), chlorogenic acid (12.4 μg/g), kaempferol-3-*O*-rutinoside (11.3 μg/g), quercetin-diglucoside (66.0 μg/g), and rutin (42.0 μg/g) [242]. As GB polysaccharides (GBPS) are major contributors of health-endowing activities and have been vastly investigated, this section will primarily focus on GBPS. GBPS are considered to be therapeutic in alternative medicine with immunomodulation, antioxidant, neuroprotection, anti-tumor, antidiabetic, radioprotection, anti-osteoporosis, hepatoprotection, and anti-fatigue activities. The GBPS biological activities depend on their molecular weight, chemical structure, and chain conformation [154,301]. The GBPS are among a few plant-based bioactive compounds that have shown simultaneous hypoglycemic and hypolipidemic properties. Due to hypoglycemic and anti-hyperlipidemic properties, GBPS may be a potent T2DM inhibitor, delaying disease prognosis, even after disease development. Antidiabetic assays showed impressive lipid and glucose reducing effects [155,302]. Acidic GBPS treatment in rat insulinoma cells decreased oxidative stress biomarkers and increased antioxidant enzyme systems. GBPS treatment of IR alloxan-treated-HepG2 cells protected against oxidative stress and improved cell survival and proliferation [302]. Similarly, the GBPS was further checked for possibly hampering glucose uptake in the gut and intestine. The GBPS intensively reduced glucose absorption in a dose-dependent manner by competing for intestinal absorption [303]. Rat insulinoma cells incubated with GBPS rescued damaged pancreatic cells, improved the survival rate, and encouraged insulin secretion. The IR cell model was supplemented with purified GBPS, which upregulated glucose consumption. GBPS was easily translocated and transported across the Caco-2 intestinal cell membrane through the SGLT-1 transporter, producing a hypoglycemic effect. Therefore, GBPS is a plant-based bioactive compound that shows simultaneous hypoglycemic and hypolipidemic properties [303]. Purified GBPS fractions showed dose-dependent hypoglycemic activities, resulting in increased glucose uptake [156,303]. Besides GBPS, GB carotenoids have also shown antidiabetic and α-glucosidase and α-amylase enzyme inhibitory activities [242].

The hypolipidemic effects of GB intake have been studied by in vivo approaches (Table 1), but human clinical trials for GB have been limited, with most studies performed using small sample sizes in China. GB consumption effectively reduced serum lipid peroxide species in diabetic patients. Reductions in waist circumference, TG, transaminase, and TC levels were reported in metabolic syndrome patients following routine GB intake. Lipid profile improvements were accompanied by increased GSH and CAT enzymatic activities [167]. GB anthocyanins reduced BW gain (17.4 to 38.7%) by increasing fecal fatty acid contents and downregulating IL-6, TNF-α, IFN-γ, NF-κB, and iNOS gene expression [157]. GBPS decoction treatment of alloxan-induced, diabetic, obese rabbits effectively reduced blood glucose levels. GBPS substantially decreased serum TG (−4.27%), TC (−3.5%), LDL-C levels, and increased HDL-C serum levels (0.78) [154]. The hypoglycemic and hypolipidemic effects of GBPS were later confirmed by the works of Zhao et al. [158]. Supplementation of HFD with GBPS decreased HOMA-IR, fasting and postprandial insulin and glucose levels, serum TG, TC, and LDL-C levels, and weight gain [158].

The oxidative stress relieving effect of GBPS was also checked (Table 1). The effect of GBPS treatment on the kidneys of STZ-induced diabetic rats increased kidney antioxidant enzymes including CAT, SOD, GBPx, GST, and GSH [170]. The supplementation of GB in the form of GB milkshakes increased plasma zeaxanthin and antioxidant levels by 57 and 26%, respectively. GB juice (GBJ) also increased GSH peroxidase (GSH-Px) and SOD by 9.87% and 8.7%, respectively and decreased MDA levels by 5.95% [166]. GBPS intake also protected against glaucoma, which was confirmed in retinal ganglion cells, and disrupted intraocular pressure [159]. GBPS administration to C57BL/6 mice reversed oxidative stress, dyslipidemia, and diabetic changes. GBPS administration downregulated nitrotyrosine and MDA expression and increased antioxidant enzymes such as CAT, GPx, and Cu/Zn SOD. GBPS intake also diminished pro-inflammatory biomarkers including TNF-α, IL-1β, iNOS, and COX-2. Following pro-inflammatory marker reduction, liver injury biomarkers, called chemokines, were also reduced. The liver regeneration process was also observed following GB intake, enhancing liver regeneration biomarkers [168,304].

With respect to hypoglycemic effect specifically (Table 1), Zhao et al. [160] confirmed the antidiabetic characteristics of GBPS, which increased GLUT-4 expression in the skeletal muscle plasma membrane. Purified GBPS in pancreatic cells increased glucose uptake and metabolism, insulin secretion, and proliferation. The enhanced glucose metabolism mechanism was associated with increased hepatic hexokinase and pyruvate kinase expression/activity (Table 1) [26,161]. GBPS may block the ATP-sensitive K^+^ channel, activate glycogen synthetase and insulin-like growth factor, enhance peripheral glucose utilization, or inhibit glucagon releasing factors in pancreatic α-cells [197]. In a recent single meal challenge study, increased glucose and lipid consumption were observed in GB-treated patients, associated with increased respiratory quotients, oxygen usage, and carbon dioxide release. However, no single-dose effects on substrate oxidation and postprandial-energy-expenditure were reported [169]. Du et al. [162] compared GBPS with metformin and reported similar normalization effects on blood glucose and insulin levels. This study also reported reduced IL-2, IL-6, TNF-α, intercellular adhesion molecule-1 (ICAM-1), MCP-1, and blood urea/nitrogen levels, inhibited albuminuria, and reversed histopathological alterations. GBPS treatment in HFD/HCD-fed rats also demonstrated hypoglycemic and hypolipidemic effects [115]. Ni et al. [163] examined the potential neuroprotective effects of aqueous GB extracts. Retinal apoptosis causes photoreceptor degradation and diabetic retinopathy (DN), and GB carotenoid supplementation in rats hampered caspase-2-induced apoptosis, protecting photoreceptors [163]. Prolonged or chronic hyperglycemia downregulates luteolin and zeaxanthin-metabolizing gene expression, causing retinopathy. GB carotenoids protected against diabetes-induced retinopathy. GB supplementation upregulated carotenoid metabolism genes and retina biogenesis in STZ-induced diabetic rats [300]. GB also contains taurine, a non-essential amino acid, and GB-derived taurine enhanced PPAR-γ activity and elevated cAMP levels, hampering the prognosis of DN with reversal of epithelial barrier impairments [300].

GBPS, polyphenol, and carotenoid effects on the gut microbiome were also studied (Table 1). Fermentation and simulated digestion experiments revealed that GBPS was digested and degraded only in the distal gut, releasing monosaccharides and promoting beneficial SCOA-producing bacterial growth. Monosaccharides with side chains are more susceptible to degradation than monosaccharides with linked backbones. GBPS greatly increased SCFA-producing gut microbiota and increased Bacteroidetes (including *Prevotella* and *Bacteroides*) and Actinobacteria (containing *Collinsella* and *Bifidobacterium*) populations, whereas *Megamonas* and *Megasphaera* (Firmicutes) populations were decreased. Furthermore, SCOA/SCFA-producing, prebiotic-effect-giving, proteolytic microflora such as *Bacteroides*, *Phascolarctobacterium*, *Bifidobacterium*, *Prevotella*, *Clostridium XlVb*, *Oscillibacter Collinsella*, and *Lactococcus* were prominent following GBPS treatment [305]. In another study, dietary GB supplementation also increased health-promoting secondary metabolite and SCOA-producing *Actinobacteria*, *Lachnospiraceae*, *Clostridium* XIVb, *Sporobacter*, *Pseudoflavonifractor*, *Butyriccicoccus*, *Anaerotruncus, Anaerosporobacter,* and *Ruminococcaceae* populations without affecting *Akkemansia*, *Mucispirillum*, *Bacteriodes*, and *Desulfovirio*. Butyryl-Coenzyme A CoA transferase is an important butyrate gene, and GBPS supplementation increased its expression in butyrate-producing bacteria such as the *Clostridium cluster* XIVa group including *Lachnospiraceae*, *Faecailbacterium prausnitzii,* and *Ruminococcaceae* [164]. The GBPS prebiotic effects increased the populations of *Firmicutes*, *Akkermansia*, *Proteobacteria*, *Lactobacillus*, and *Prevotellaceae* [165].

## 12. Acai Berries

Acai berry (AB, *Euterpe oleracea*) is native to South America and has high phytochemical contents. The dominant antidiabetic phenolic acid constituents in AB include ferulic acid, anthocyanin-3-glycosides, *p*-hydroxybenzoic acid, epicatechin, protocatechuic acid, gallic acid, ellagic acid, catechin, *p*-coumaric acid, vanillic acid, and gallotannins (Table 2) [246]. Anthocyanin and flavonoids are prominent therapeutic polyphenols including C3G and C3R [244,245]. AB juice (ABJ) is richer in polyphenols and flavonoids than other berry juices, resulting in increased antioxidant capacities [200]. In in vitro studies, the isotonic ABJ pancreatic lipase inhibitory activity was significantly positively correlated with anthocyanin contents. Isotonic ABJ also reduced adipogenesis and lipid accumulation in 3T3-L1 adipocytes and inhibited α-glucosidase activity [306]. Isotonic ABJ also inhibited Cu-mediated LDL oxidation and oxidized or acetylated LDL uptake. AB puree also showed antiglycation activities at a concentration 0.1 mg/mL, which was 89% stronger than the control [171]. Polyphenols in ABJ affect adipogenesis, preventing obesity, weight gain, inflammation, and diabetes [307].

In in vivo studies, AB fruit proved to be a very useful therapeutic agent for circumventing oxidative stress, and controlling dyslipidemic and metabolic syndrome conditions (Table 1). The supplementation of AB fruit effectively prevented protein oxidation as increased protein sulfhydryl groups were observed, with decreased protein oxidation biomarker carbonyl proteins. A single AB pulp dose enhanced plasma antioxidant capacity 7-fold 3 h after its consumption. Plasma anthocyanins reached maximum levels 2.2 h after AB pulp consumption [179,180]. In another in vivo study, AB pulp supplementation in oxidatively damaged mutant *Drosophila melanogaster*, in combination with HFD, reversed HFD-induced oxidative stress damage and prolonged the lifespan expectancy by 22% [172,308]. AB supplementation with exercise improved hepatic oxidation status by reducing inflammatory MCP-1 expression, SOD activity, redox-sensitive signaling pathway activation, ROS generation, and ROS stress [173]. To elucidate the antidiabetic and antioxidative molecular mechanism of AB, AB-mediated transcript-level changes were examined in 12 genes associated with JNK, nutrient sensing, and insulin-like signaling pathways [309]. PEPCK genes, involved in glyceroneogenesis and gluconeogenesis, were reduced in the AB pulp group. Cholesterolemic diet consumption decreased lethal/essential or life gene (lefl2) expression, which was reversed by AB fruit consumption. Two JNK targets, metallothionein A, and glutathione S transferase D1, which have antioxidant activities, were upregulated after AB consumption without affecting the remaining JNK downstream target genes (Ferritin 1 heavy chain homolog, Ice, Heat shock protein 68, and Puckered). Moreover, AB ingestion promoted longevity by intensifying stress response pathway activity and suppressing PEPCK genes [172,309]. Treatment with AB seed extracts also reduced blood pressure, the hypertension biomarker renin, and DN biomarker levels (creatinine, urea, creatin, and albumin). Diabetes onset leads to oxidative stress and hypertension, decreasing the number of glomeruli per area per kidney, a major DN marker. AB seed extracts reduced kidney volume expansion and prevented a decrease in the number of glomeruli per area per kidney [174]. AB seed extracts substantially reduced renal injury (resulting in reduced urea and creatine excretion), hampering renal fibrosis progression. The diabetes-induced glomerular filtration barrier injury markers, podocin and nephrin, decreased in diabetic male Wistar rats, whereas AB seed extract treatment restored these levels. AB seed extract treatment also reduced renal proinflammatory cytokines and oxidative stress biomarkers, reinforcing the anti-oxidative defense system [174]. The effects of exercise and AB seed-rendered extract consumption in STZ and HFD-induced diabetic rats reduced HbA1C, glycemia, serum insulin, HOMA-IR, serum TG, TC, LDL-C, and HDL-C levels [176]. Insulin signaling components (insulin receptors, pAKT, and AKT) in skeletal muscles were upregulated following AB seed extract consumption and exercise [176]. Reduced adiponectin levels are observed in T2DM, associated with deregulated sugar and lipid metabolism, and AB seed extracts reversed this effect. AB seeds induced increased GLUT-4 expression and glucose uptake due to AMPK activation [176] and increased GLP-1 and incretin levels with reduced leptin and inflammatory cytokine expression, which were not observed in HFD-fed rats treated with exercise alone. Increased GLP-1 and incretin expression promotes insulin secretion, suppressing gastric emptying, and glucagon synthase [176,310]. The same research group then used the AB seed extracts to check the anti-obesity features in the C57BL/6 mice strain fed on HFD. HFD supplemented with the AB seed extract prevented weight gain in mice [311]. Adiponectin levels, which are responsible for lipid metabolism, decreased in HFD-fed mice and were restored by AB seed extract supplementation. AB seed extracts increased glucose and lipid metabolizing protein expression including pAMPK/AMPK, pACC/ACC, HMG-CoA, and various transporters including ATP-binding cassette sub-family G member 5-ABCG5 and ATP-binding cassette sub-family G member 8-ABCG8, while reducing SREBP-1c expression. Similarly, protein and lipid oxidation products including carbonyl proteins and MDA were reduced by strengthening the anti-oxidative enzyme system [311].

Regarding glucose-lowering effect, recently, the human AB fruit consumption with normal meals decreased FBS and mean plasma insulin levels after one month. Plasma TG, TC, and LDL-C levels, and the LDL-C/HDL-C ratio also decreased, with increased plasma HDL-C levels [181]. The AB consumption with HFD enhanced fecal cholesterol contents, with no influence on low-grade-inflammation biomarkers [113]. Freeze-dried AB fruit pulp reversed the HFD-induced alterations in PEPCK expression [312]. Aqueous ethanolic AB extracts restored mitochondrial complex I function by modulating NADH:ubiquinone oxidoreductase core unit 7 and 8 expression. NLRP3 (a component of inflammasome) and caspase 1/caspase 3/caspase 8 (Interleukin-1 converting enzyme family, which initiates inflammatory response) were downregulated in oxidative-agent-treated macrophages [313]. AB supplementation also interfered with hepatic cholesterolemic metabolism. AB attenuated the high-cholesterol diet effects by reducing weight gain, TC and LDL-C levels, and key regulatory gene expression associated with the cholesterol biosynthesis pathway including HMG CoA-R, EBP-2, ApoB100, LDL-R, ABCG8, and CYP7A1 [175]. Intensive feeding with freeze-dried AB pulp attenuated HFD-induced hepatic steatosis by improving IR, adiponectin expression, adiponectin receptor 2, SREBP-1c, PPAR-α, and its target gene, CPT. Fat accumulating gene expression including UCP-2 and fatty acid translocase were reduced by AB treatment [179]. Both lipid accumulation and oxidation were reduced in zebrafish fed with a high-cholesterol diet, and reduced serum TC, LDL-C, and MDA levels were observed in AB-treated zebrafish [171]. Aside from lipid oxidation inhibition, the AB intake also prevented amino acid oxidation after HCD, reducing protein carbonyls and sulfhydryl groups, which are important protein damage biomarkers. Reduced arylesterase and PON activities and reduced hepatic ALT, AST, and ALP levels demonstrated improved hepatic operation [175]. AB powder also improved anti-inflammatory mechanisms after HFD by improving glucose intolerance and reducing IL-6 and TNF-α concentrations in epididymal adipose tissue [312].

A comprehensive study examining AB intake on the gut microflora is currently lacking. Simulated digestion studies examining AB polyphenols inhibited the growth of symbiotic and saccharolytic *Bacteroides*, *Prevotella*, and *Clostridium histolyticum*. AB polyphenols showed favorable effects on the intestinal SCFA bacteria population including LAB [178]. Guergoletto et al. [177] noted increased intestinal populations of obesity-protecting bacteria (i.e., *Bifidobacterium* spp., *Eubacterium rectale–Clostridium coccoides* group, *Bacteroides spp—Prevotella group,* and *FOS-Raftilose* P95). However, AB polyphenols showed no considerable effects on *Enterococcus spp* and *C. histolyticum* [177].

Conclusively, AB exerted antidiabetic, anti-obesity, antioxidative, and anti-inflammatory actions by reducing the expression of PPAR-γ and its modulators (C/EBP-ß, C/EBP-δ, and other C/EBP family members, Kruppel-like factor, and SREBP1C) Moreover, decreased expression level of transcriptomic factors such as C/EBPß (−0.41%), C/EBPα (−0.66%), Kruppel like factor (−0.83%), and SREBP1C (−0.24%) were also seen [125,133]. AB also reduced the expression levels of lipogenic genes FAS (−0.5%), aP2 (−0.7%), LPL (−0.7%), and FATP1 (−0.55%). Low-grade-inflammation biomarkers including leptin and total PAI decreased with increasing anti-inflammatory and anti-adipogenic adiponectin levels [170,172,309,314]. The expression levels of the pro-inflammatory factors NF-κB, TNF-α, MCP-1 (−0.81%), IL-6 (−0.48%), IL-8 (−0.05%), IL-1βß (−0.03%), and INF-β(−0.49%) were also reduced. TNF-α activates NF-κB and interleukins (IL-2 and IL-6), which was prevented by ABJ polyphenols [170,313,314].

## 13. Chokeberries

**Figure 2:**
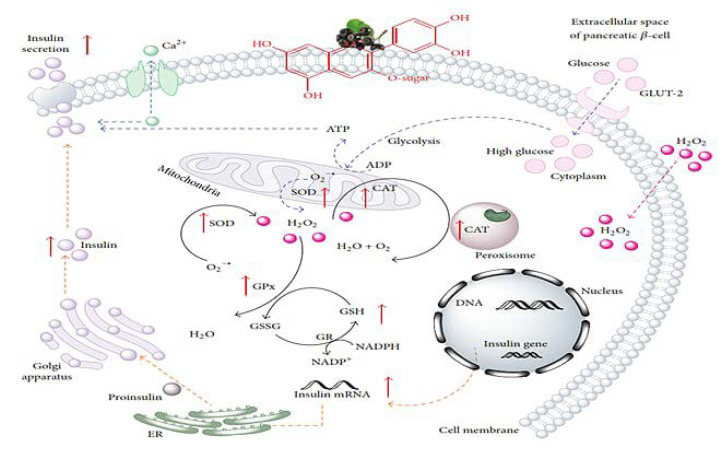
Schematic presentation of chokeberry anthocyanin-induced insulin secretion and antioxidant enzyme pathways in pancreatic β-cells under high-glucose-induced stress conditions. Glucose is transported across the cell membrane via glucose transporter (i.e., GLUT-2), followed by glycolysis and pyruvate production. Afterward, pyruvate is used for the generation of ATP in mitochondria. Here, in connection with the electron transport chain, radicals, like superoxide anion (O_2_^•‒^), are also produced and simultaneously neutralized by the enzymatic antioxidant SOD. SOD converts the O_2_^•‒^ into harmless O_2_ and another radical H_2_O_2_. In addition to H_2_O_2_ diffusion through the cell membrane, H_2_O_2_ is also scavenged by CAT and GPx resulting in water and oxygen production. Chokeberry-derived anthocyanins strengthen this inherent enzymatic antioxidant system (i.e., SOD, CAT, and GPx), which can more actively neutralize the radicals generated during glucose metabolism. H_2_O_2_-stimulated reduction of GSH is also ameliorated by chokeberry anthocyanins. Chokeberry anthocyanins also replenish the pool of insulin by increasing the insulin gene expression. Proinsulin, a precursor of insulin, folded in the endoplasmic reticulum, is transported to the Golgi apparatus. Chokeberry anthocyanins can also influence the opening of the voltage-gated Ca^2+^ channels, leading to an increased fusion of insulin granules with the cell membrane (Source: Rugina et al. [316]).

Chokeberries (black chokeberry (BCB), *Aronia melanocarpa*, red chokeberry (RCB), *Aronia arbutifolia*) can be consumed as whole fruit, jam, wine, juice, syrup, tea, soft spreads, chili starters, salsa, beer, extracts, gummies, ice cream, and tinctures. CB consumption was used to treat colds in America and to treat hyperglycemia, metabolic syndrome, and hypertension in Europe and Russia. In in vitro bioassays, CB extract (CBE) showed significant α-glucosidase inhibitory activity compared with the referenced antidiabetic drug acarbose. Purified anthocyanins (cyanidin 3-galactoside, cyanidin 3-arabinoside, cyanidin 3-glucoside, and cyanidin 3-xyloside) were the strongest antidiabetic compounds compared with isolated dimeric and trimeric procyanidins. BCB juice (BCBJ) also inhibited α-glucosidase, dipeptidyl peptidase (DPP) IV, and ACE activities by 75, 35, and 95% in a dose-dependent manner, respectively [182]. BCB fermentation and digestion increase polyphenol bioaccessibility. Fermented and digested *Aronia* kefir showed stronger α-glucosidase (IC_50_ = 152.53 ± 15.24 mg kefir/mL) and pancreatic α-amylase inhibitory (IC_50_ = 146.52 ± 5.37 mg kefir/mL) activities than non-fermented *Aronia* (IC_50_ = 365.16 ± 370 48.84 mg and 196.21 ± 5.50 mg, respectively) [315]. BCBJ relieved oxidative stress in βTC3 cells by restoring the anti-oxidative enzyme pool and insulin secretion, as comprehensively explained in Figure 2 [316]. The oxidative-stress-induced reduction in insulin secretion was restored by the BCB extract (BCBE) treatment under basal glucose conditions [316]. BCBE treatment of pancreatic cells nullified cytokine (IL-1β and IFN-γ)-induced effects and decreased oxidative stress production [183]. BCBE pretreatment (0.001, 0.01, 0.1, or 1 mg/mL) of diabetic hepatic cells line RINm5F) reduced cytokine-induced-oxidative stress from 19.3–0.39 µM to 14.9–0.35 µM [183]. Similarly, BCBE pretreatment of HAECs nullified the TNF-α-induced ICAM-1 and VCAM-1 expression by 35 and 45%, respectively, in a dose-dependent manner. BCBEs also prevented NF-κB p65 phosphorylation, which activates the pro-inflammatory transcription factor NF-κB [317,318].

Addressing the anti-inflammatory potential of CB, in in vivo clinical studies, Kardum et al. [195,196] administered CBJ to patients with pharmacologically incurable grade I hypertension and high blood pressure, resulting in decreased systolic/diastolic blood pressure, with a stronger effect associated with long-term consumption. CBEs also reduced systolic/diastolic blood pressure [197], particularly in congenital heart disease patients [198]. Following hypertension, inflammation is another diabetes complication and numerous studies have cited the anti-inflammatory potential of BCB or its juice consumption. Increased PPAR-γ2 expression was attenuated by BCBEs, reducing downstream lipid metabolizing PPAR-γ2 target expression such as PGE receptor and LPL, decreasing intracellular lipid droplet accumulation [184]. Regular BCBJ consumption improved chronic inflammatory conditions, lowering IFN-γ and TNF-α levels [195,196,198]. The immunomodulatory effects of BCB intake have also been discussed in the literature in STZ-induced male Wistar rats. DM causes immune imbalances because damaged pancreatic cells trigger macrophage and T lymphocyte infiltration, which lesion β-cells. BCB consumption by STZ-induced male Wistar rats reduced fibrinogen, TNF-α, and IFN-γ levels, which returned to their normal values 72 h post-administration of BCB [199].

Regarding hypoglycemic response, BCBJ consumption also modulated circulating lipid levels including TG, TC, and LDL-C in mild hypertensive patients (Table 1) [185,198]. BCBJ consumption also reduced serum TG, TC, and LDL-C levels in hypercholesterolemic healthy subjects [128]. Long-term BCB consumption was recommended for desirable hypoglycemic and hypolipidemic effects [128,185,198]. Valcheva-Kuzmanova et al. [186] demonstrated up to 39% reduced postprandial serum TG levels in STZ-induced diabetic rats after BCBJ consumption and reported encouraging results for both diabetic and healthy rats. However, Lipińska and Jóźwik [187] showed pronounced hypolipidemic effects only in diabetic Polish Merino lambs including significantly decreased serum LDL-C and increased HDL-C levels, without significant effects on serum TC levels. In addition to preventing increased plasma glucose, homocysteine, and fibrinogen levels, reduced serum lipid levels (TG, TC, and LDL-C) were observed in STZ-induced diabetic rats [201]. Hepatic steatosis and NAFLD were prevented by BCB treatment in HFD-fed diabetic C57BL/6N mice. Daily BCBE administration prevented increased body, liver, and epididymis weights [188]. Several possible mechanisms have been proposed in the literature referring to the lipid-lowering property of BCB consumption. The BCB hypoglycemic effect may be associated with increased cynidine-induced lipid metabolism, reduced catechin-induced cholesterol absorption, and the flavonoid-influenced downregulation of cholesterol synthesis enzymes including HMG-CA-R, cholesterol acyltransferase, and acyl-CoA [185,188].

The anti-oxidative, anti-obesity, and anti-diabetic potential of BCB was checked in the various diabetic model mice (Table 1), where BCB increased serum insulin secretion with reduced pro-inflammatory cytokine expression (MAPKs, NF-κB, COX-2, and iNOS) in a dose-dependent manner [183]. Jurgoński et al. [189] fed BCBE to high-fructose-diet-fed STZ-induced diabetic rats and showed increased maltase and sucrase activity, and decreased lactase production in the small intestinal mucosal membrane. Daily BCBJ consumption lowered postprandial glucose levels after OGTT, regardless of gender, and reduced ACE, α-glucosidase, and DPP IV activities in a dose-dependent manner [182]. Valcheva-Kuzmanova et al. [186] showed lower postprandial glucose levels (up to 44%) in STZ-induced diabetic rats after BCB consumption, and Lipińska and Jóźwik [187] demonstrated a pronounced FBS decrease in BCB-treated Polish merino lambs. Postprandial OGTT results for BCB-treated mice decreased, with improved intraperitoneal ITT results [185]. Similarly, consumption by STZ-induced diabetic mice reduced serum TBARS levels and mitigated lipid peroxidation (by 29–50%) and kidney hypertrophy [190]. Following CCl_4_ administration, the decreased concentration of CAT, GPx, and GR were increased by 117%, 56% and 44%, respectively, after the intake of BCBJ. Protein carbonyls, protein oxidation biomarkers, decreased by 22% after BCBJ consumption in male Wistar rats [190]. BCBJ consumption by the KK-Ay and C57BL/6JmsSlc mice reduced BW, white adipose tissue weight, α-glucosidase and DPP IV activity, and blood TG levels. Mesenteric, epididymal, subcutaneous, and retroperitoneal white adipose tissue weights were reduced by 26%, 27%, 48%, and 38% compared with those in control animals [39]. Bhaswant et al. [191] administered BCBJ to male Wister HFD- and HCD-fed rats and observed reduced BW gain and feed conversion efficiency. Total body fat mass, BMI, abdominal fat (epididymal, omental fat pads, and retroperitoneal), and visceral adiposity index reductions were more pronounced in Wistar rats fed with BCBJ than in those fed with biofunctional purple maize flour. BCBJ consumption also reduced liver injury biomarkers (ALP, AST, and ALT), although these levels remained within the normal range [191]. In another study, male Wistar rats were fed high-fructose diets containing BCBE, resulting in increased plasma HDL-C and adiponectin levels [192]. IRS-1/2 and PI3K regulatory subunit protein expression increased by 2.3-, 1.8-, and 1.5-times, respectively, along with inhibiting the phosphatase and tensin homolog (Pten) (−0.61%) expression. The expression level of glucose uptake, transportation (GLUT1 and GLUT4) and gluconeogenesis (GYS) was uplifted by 1.5 times compared to high-fructose fed control rats. BCB consumption inhibited lipogenesis and lipid accumulation by reducing fatty acid-binding protein, FAS, and LPL (lipogenesis protein) by 0.6–0.7%. Improved glucose and lipid metabolism and increased glucose and lipid regulatory metabolizing protein expression (adiponectin and PPAR-γ) were also observed [192]. Cynidine 3, 5-diglucoide was identified as a DPP IV inhibitor. DPP IV cleaves incertins including GLP-I and glucose-dependent-insulinotropic polypeptide at their N-terminal regions, resulting in decreased insulin secretion [182,186]. Cyanidin glycosides including 3-galactoside, 3-glucoside, cyanidin 3-*O*-β-glucoside3-arabinoside, and 3-xyloside enhance glucose uptake and GLUT4 translocation. Diabetes-associated hyperlipidemic complications were improved by regulating the FOXO1-mediated adipose TG lipase transcription [185].

BCB contains high levels of anthocyanins (1958.18 mg/100 g FW), proanthocyanidins (522–1002 mg/100 g FW), and hydroxycinnamic acids (187.9 mg/100 g FW) including chlorogenic acid and neochlorogenic acid [212,247]. Cynidine-3-*O*-glucoside, cynidine-3-*O*-galactoside, cynidine-3-*O*-xyloside, and cynidine-3-*O*-arabinoside are the primary antidiabetic and anti-oxidative anthocyanin compounds in BCBJ (Table 2). No studies have examined the CB consumption effects on gut microflora in diabetic/obese individuals, although CB consumption has been examined in healthy individuals [202]. Chronic BC capsule treatment influenced the intestinal diversity of health promoting and SCOA-producing *Anaerostipes*, *Bifidobacterium*, *Faecalibacterium*, and *Clostridium* genera. CBE capsules increased the relative abundance of *Anaerostipes*, whereas whole CB capsules increased *Bacteroides* and *Clostridium* XiV populations. Correlation analysis between gut microbial genera and plasma polyphenolic contents revealed that *Prevotella*, *Dialister*, *Desulfovibrio,* and *Bifidobacteria* were responsible for the increased levels of nine, eight, seven, and six health promoting plasma CB metabolites, respectively, including derivatives of benzoic acid, hippuric acid, phenylacetic acid, cinnamic acid, caffeic acid, flavonols, (iso)ferulic acid, benzaldehydes, and pyrogallol [202].

## 14. Black Currants

Black currant (BCT, *Ribes nigrum* L.) is cultivated primarily in Europe, New Zealand, and Australia. BCT is a rich source of anthocyanins that represent 95% of polyphenolic compounds, with the remaining 5% including other minor polyphenol classes. Delphinidin-3-rutinoside (D3R) is the major BCT antidiabetic anthocyanin compound that improves glucose tolerance. In BCT nectar, cynidine and delphinidin rutinosides are the dominating anthocyanins, followed by glucoside compounds [93,319]. A full list of other therapeutic BCT compounds are presented in Table 2. GLP-1 and AMPK are the primary BCT polyphenolic compound targets. BCT extract (BCTE) consumption increased GLP-1 secretion. GLP-1, an incretin, promotes pancreatic β-cell division and glucose-dependent insulin release [212,213,289]. BCTEs contain approximately 70% anthocyanins (especially rutinosides and glucosides of delphinidin and cyanidin) and are considered to be effective α-glucosidase inhibitors [289]. Apple and BCT juice (BCTJ) treatment in human Caco-2 cells reduced sodium-independent and total glucose uptake by 46 and 51%, respectively. In oocytes, apple and BCTJ-derived phloretin and phlorizin effectively reduced glucose uptake by 58 and 85%, respectively [213]. The BCT polysaccharide BCP-I also showed remarkable antiglycation activities due to its inhibitory effects on Amadori products [320]. BCT powder incorporation into high-glycemic-indexed food decreased glucose release and increased antioxidant capacities [321].

In addition to in vitro studies, glucose and lipid lowering effect of BC extracts or its screened anthocyanins have also been investigated enormously in various in vivo studies (Table 1). The intake of major BCT anthocyanin consumption, in combination with intraperitoneal glucose administration, prevented increased serum glucose concentrations with the simultaneous increase in serum insulin levels [203]. Improved hyperglycemia and hypoinsulinemia are caused by the GLP-activation-induced increase in insulin secretion. BCT powder, administered for six days before OGTT, improved postprandial plasma insulin and glucose levels in healthy human subjects [214]. BCTE consumed with a normal diet by KK-Ay mice induced hypoglycemia and modulated basal GLP-1 concentrations without affecting plasma insulin levels, food intake, or BW [204]. Proglucagon cleaving agent proprotein convertase subtilisin/Kexin type 1, which processes proglucagon into GLP-1, increased. BCTE treatments also increased AMPK phosphorylation in skeletal muscles, upregulating insulin-independent glucose uptake pathways by increasing downstream target expression including GLUT-4 and the translocating plasma membrane [204]. Previously, Esposito et al. [205] also conducted an anti-diabetic study using 1% BCT powder, which decreased rat BWs, irrespective of dietary fat contents. Microbiological fecal analyses showed increased fecal anthocyanin contents, especially in lean animals. These results suggested that gut microflora more actively transform polyphenolic metabolites in lean animals rather than in obese animals. BCT supplementation reversed the postprandial glucose levels associated with HFD; however, the postprandial glucose level continued to rise due to gut microbiota disruption. Similarly, BCT improved HFD-induced insulin, but the gut microflora disruption increased IR. These results signified the importance of gut microflora during the BCT polyphenol metabolization and biotransformation [205]. The supplementation of 0.1% BCTE in HFD reduced retroperitoneal and epididymal adipose fat. BCTE hypolipidemic characteristics were verified by upregulated lipogenic/lipid metabolizing genes in adipocytes including UCP-2, UCP-3, mitochondrial transcription factor A (TFAM), PPAR-α, SREBP-1c, FAS, and SCD-1, and fatty acid oxidation genes including CPT-1α and 1β [206]. Repressed inflammatory marker expression in macrophages has also been reported. Reduced IKKε (an enzyme complex that is involved in propagating the cellular response to inflammation) and TANK-binding kinase 1 (a member of IKK subfamily, which activates in response to lipopolysaccharides) expression was observed in the BCT-treated group, compared with upregulation in the HFD group [206,212]. BCTJ/nectar waste extract (pomace) was much richer in anthocyanins than in BCT pulp. Phytochemically, BCT pomace extracts are rich in D3G, D3R, cyanidin-3-rutinoside, glycosides, and flavonol aglycones. HFD supplemented with BCT pomace extracts did not affect food intake or BW. Fat in the diet increases small intestinal digesta viscosity, whereas BCT pomace polyphenolic extracts made this digesta more acidic [207]. The polyphenolic-rich BCTE also reduced cecal tissue mass and increased ammonia contents. HFD reduced bacterial glycolytic enzyme activities such as α- and β-galactosidases and α- and β-glucosidases, which were restored by BCT pomace extract. BCT supplementation reduced β-glucuronidase activity, which is associated with reduced pressure on the intestinal detoxification mechanism [208]. BCT supplementation reduced the cecal putrefactive SFCA concentration, regardless of diet [207,208]. BCTE consumption increased mean fat oxidation during prolonged cycling exercise by endurance-trained females with reduced mean carbohydrate oxidation [215]. However, the opposite outcome was observed when BCTJ was consumed before exercise, without significant effects on blood lactate, glucose, and MDA levels [216].

In addition to HFD, the high-fructose-diet or HCD were also involved in the hyperglycemic, hyperlipidemic, and metabolic syndrome conditions. BCTE administration with high-fructose-diet prevented increases in liver weight, BW, and epididymal fat pad weight. OGTT results improved, with decreased p-AMPK and IRS-1 levels in the BCTE-treated group. BCTE supplementation also decreased high-fructose-diet-induced hyperglycemic marker expression and reduced atherosclerosis risk by diminishing ICAM-1, VCAM-1, E-selectin, endothelin, and eNOS expression levels in aortic tissues [209]. Consumption of an anthocyanin-rich sugar-free BCT drink with a normal-carbohydrate diet delayed the glycemic and insulinemic response with reduced incretin and GLP-1 expression [212,213]. The consumption of BB, BCT, CrB, and strawberries restricted post-meal blood insulin and glucose fluctuations induced by HFD/HCD. LB combined with BCT (whole or nectar) ameliorated postprandial insulinemic and glycemic control and response [62,123,267]. The irreversible hydrolysis of sucrose into fructose and glucose under high temperature and low pH conditions produces invertase sugars. BCT nectar, sweetened with invertase sugars, reduced postprandial blood glucose levels and the maximal blood glucose level by 33 and 87%, respectively. The nectar x time interaction also revealed lower insulin secretion at 15 and 30 min of post-nectar-consumption and expulsion of insulin from the baseline was cut by 13% compared to the reference [62,123,267].

Regarding oxidative stress and diabetes-related complications, ample amounts of evidence have suggested that anthocyanins from BC exert anti-hypertensive, anti-inflammatory, anti-fibrotic, and anti-hepatic steatosis effects by limiting lipogenesis and gluconeogenesis (Table 2) [217]. BCT-derived purified extracts administered to hepatic steatosis model C57BL/6J mice did not prevent BW loss, but serum ALT and AST levels increased. BCT anthocyanin supplementation decreased hepatic TG and TC accumulation [304]. Histological analysis showed that microvascular steatosis, inflammatory cell infiltration, and hepatocyte ballooning were reduced by (up to 50%) BCT anthocyanins. Hepatic stellate cells produce collagen during fibrogenesis. Reduced α-smooth muscle actin and upregulated carbamoyl phosphate synthase 1 suggest hepatic stellate cell inhibition, inhibiting fibrosis and non-alcoholic hepatic steatosis. BCTE treatment increased mitochondrial biogenesis and decreased the AMPK/pAMPK ratio and pivotal mitochondrial biogenesis regulators including PGC-1α and β, Nrf-1 and -2, and TFAM. Mitochondrial fatty acid β-oxidation occurs due to mitochondrial oxidative phosphorylation, which was reversed through effects on PPAR-α, CPT-1, and medium-chain acyl CoA dehydrogenase expression [62,93,123,217,304,319].

## 15. Maqui Berries

Maqui berries (MB) (*Aristotelia chilensis)* have recently gained attention due to their high content of polyphenolic compounds. The stated phytochemical composition of MB was 138 ± 0.4 mg/100 g fresh weight with 35% relative abundance of delphinidin [218]. Di Lorenzo et al. [219] analyzed the MqB composition (Table 2). MqB is rich in anthocyanins including 84% diglycosylated and 16% monoglycosylated anthocyanins [251,322,323]. The in vitro sugar hydrolyzing enzymes inhibitory activities of MB extracts were reported by Rubiliar and his colleagues. Rubiliar et al. [324] reported α-amylase and α-glucosidase inhibitory activities, resulting in decreased postprandial glucose levels and improved glucose tolerance [324]. Crude and purified MqB extracts (MqBEs) reduced MDA production and minimized oxidative damage [250]. An isotonic soft drink containing lyophilized MqB, acai, and blackthorn berry powders [152] demonstrated pancreatic lipase and α-glucosidase inhibitory activities, which were superior to the control, acai-, and blackthorn-based beverages. Likewise, the in vitro anti-diabetic assay showed the inhibition of α-glucosidase activity by 90% compared to the lemon juice control (80%), whilst the recorded inhibitory α-glucosidase activity of tested commercial isotonic drinks was around 50% [306]. The MqBE anti-diabetic and anti-lipidemic potentials were further examined in RAW264.7 mouse monocytes and 3T3-L1 mouse pre-adipocytes [220,325]. MBEs reduced adipocyte formation by promoting MMP-2 and MMP-9 (endopeptidases). GST treatment decreased GSH, SOD, and CAT expression, which was reversed by MqBE treatment in macrophages. LPS treatment increased IL-6, MCP-1, TNF-α, and galectin-3 with decreased adiponectin expression, which was countered and reversed by MqBEs in macrophages [220]. Furthermore, a dose of 100 and 180 µM MqB delphinidin inhibited sodium palmitate-induced-TG-accumulation by 50 and 59%, respectively, in Hep2G cells [222].

With respect to in vivo antidiabetic and anti-obesity potential of MqB (Table 1), Rojo et al. [220] fed C57BL/BJ mice anthocyanin-rich MqBEs, which significantly decreased plasma glucose levels following glucose ingestion. Anthocyanin-rich MqBEs also reduced G6Pase and increased insulin sensitivity. Glucose uptake was upregulated in L6 skeletal muscle cells, without toxic effects [220]. Delphinidin 3-sambubioside-5-glucoside, a signature MqB biomarker, showed an equivalent capacity to metformin for normalizing blood glucose levels [326]. Lipid accumulation was inhibited by 4–11% by MqBE treatment in 3T3-L1 mice; however, lipogenesis was inhibited by 6–38% during adipocyte differentiation. The lipogenesis inhibitor protein, preadipocyte factor 1, was upregulated in MqB-treated 3T3-L1 mice. MqB supplementation also exerted an anti-inflammatory response by reducing ROS expression by 9.8 to 61.8%. The expression of COX-2 and production of PGE2 was also evaluated in the RAW 264.7 macrophages to understand the anti-inflammatory mechanism of MqB. MqB inhibited PGE2 expression and reduced COX-2 expression (by 16.2–62%), inhibiting LPS-induced iNOS/NO production and COX-2/PGE2 pathway activation in macrophages [218,326]. MqB delphinidin anthocyanins inhibited glucose uptake and transport from the rat duodenum by inhibiting SGLT-1. The inclusion of MqB-derived 35% anthocyanins and 25% delphinidin glycosides in a rice-chicken diet effectively reduced postprandial glucose levels. Purified delphinidin anthocyanin supplementation with a normal diet reduced fasting glucose and insulin levels [218,326]. MqB anthocyanins, in capsular form (3 × 150 mg per day), decreased oxidized LDL-C and 8-iso-prostaglandin F2α, a urinary excretion oxidative stress marker [221,326]. Furthermore, MqB-derived-delphinidin treatment effectively increased AMPK phosphorylation. Gene expression analysis showed that sodium palmitate exposure upregulated lipid accumulating genes such as SREBF1, CPT1-A, patatin-like phospholipase domain containing 2, and FASN, which were reduced by delphinidin treatment. Delphinidin supplementation limited weight gain in HFD-fed C57BL/6Nhsd mice, but not increased liver weight. Glucose homeostasis variations induced by HFD/HCD were also minimized by delphinidin treatment [222]. Hidalgo et al. [327] showed that delphinidin supplementation in rat jejunum tissues/cells reduced the short circuit current generated by glucose addition to an Ussing chamber. Delphinidin halted 3-*O*-methyl-glucose incorporation in the mouse intestine, with effects similar to the inhibition of electrogenic glucose transportation by SGLT-1 [328]. In response to delphinidin treatment and FFA1 activation, the Gαq/11 subunit was coupled with inositol trisphosphate, propionyl l-carnitine, and diacylglycerol upregulation, which modulates intracellular Ca^2+^ from the endoplasmic reticulum. In previous studies, delphinidin treatment also caused intracellular Ca^2+^ release and prevented 3-*O*-methyl-glucose uptake by FFA1 activation. Therefore, delphinidin may represent a new ligand class that can reduce intestinal glucose uptake through FFA1 activation and increased cAMP expression [327].

MqB juice (MqBJ) consumption limited oxidation in human subjects (Table 2). The copper-triggered LDL-C oxidation time lag increased with MqBJ consumption because anthocyanins chelate copper. LDL-C oxidation time is proportional to the MqBJ anti-oxidative capacity. H_2_O_2_ treatment-induced increased oxidative stress was reduced by MqBJ treatment in human umbilical vein endothelial cells [329]. A pilot study showed that the daily MqBE consumption with folic acid and berberine effectively reduced TC, LDL-C, oxidized cholesterol glycemia, free radical levels, and increased serum antioxidant capacity. Furthermore, the insulinemia, microalbuminuria, HDL, CRP, and TG values increased. MqB treatment counteracted hyperlipidemia, hyperglycemia, and ROS production in metabolic syndrome patients. An MqB polyphenol-based-nutraceutical reversed low-grade-inflammation, oxidative stress, and atherosclerogenesis in pre-diabetic patients [223]. MqBE and purified anthocyanin consumption showed positive outcomes for post-stroke stress and depression in diabetic mice. MqBEs and anthocyanins can mitigate anhedonia in humans. Anhedonic mice consumed less sucrose with increased water intake, which was mitigated by MqBE or purified anthocyanins in a dose-dependent-manner. Stroke and stress biomarkers such as TBARS, SOD, CAT, and GSH levels decreased following MqBE/anthocyanin treatment in stroke model mice [219].

## 16. Conclusions

This review aimed to collect and discuss scientific evidence regarding the positive role of berry consumption on the prevention of diabetes and its complications. Available human, animal, and in vitro studies were collected and comprehensively presented. This review demonstrated that berry product consumption represents a reliable and effective method for preventing and managing metabolic hyperglycemic and hyperlipidemic conditions. Variations in postprandial glucose and insulin levels could be reversed and normalized in diabetic subjects following post-meal berry consumption as supplements for HFD/HCD. Berry anthocyanins promoted glucose uptake and metabolism by activating pAMPK/AMPK, GLUT-4, and SGLUT-1, and inhibited weight gain and pro-inflammatory responses, downregulating lipogenesis genes (adipogenic transcription factors and PPAR-γ2) and pro-inflammatory cytokine production. Berry consumption also showed glucose-lowering and insulin sensitivity improvements, which are closely associated with hypoinsulinemia, insulin signaling activation (in adipose and skeletal muscles), the adiponectin-AMPK pathway, and GLP-1 upregulation. Regarding the relation of gut microbial ecosystem and DM, berry intake not only counteracted the deleterious HFD/HCD effects, but also favored the population of health promoting fermentative, SCOA/SCFA-producing, obesity-preventing, glycolytic, proteolytic, and secondary metabolites metabolizing microflora. The primary potential health-promoting classes of bioactive compounds found in berries include glycosides, glucosides, catechins, epicatechins, proanthocyanidins, cynidines, delphinidins, quercetin, myricetin, malvidins, petunidin, flavanols, flavonols, caffeic acids, chlorogenic acids, phenolic acids, ferulic acids, *p*-coumaric acids, vanillic acids, ellagic acids, hydroxycinnamic acid derivatives, and polysaccharides. Based on the reviewed papers, to obtain these health-endowing effects, the daily recommended dose of whole berry varies from 200 to 400 g of berry intake for a 70 kg BW middle aged person.

## Abbreviations

1-Deoxynojirimycin (1-DNJ); Acai berry (AB); acetyl coenzyme A carboxylase (ACC); acyl-CoA oxidase (ACOX); adhesion molecules nuclear factor (IκBα); advanced glycation end-product (AGE); albumin retention (AR); alcohol-free fermented blackberry juice (AFBBJ); alkaline phosphatase (ALP); aminotransferase (ALT); AMP-activated protein kinase (AMPK); angiotensin I-converting enzyme (ACE-1); apolipoprotein A (Apo)A-I; aspartate aminotransferase (AST); ATP-binding cassette (ABC); ATP-binding cassette transporter (ABCA1) Bilberry juice (BBJ); BB/BB extracts, (BBEE); Bilberries, (BBs); Black currant (BCT); BlB extracts (BlBEs); BlB juice (BlBJ); Blueberries (BlBs); body weights (BW); brown adipose tissues (BAdT); capillary albumin filtration (CAF); carbohydrate response element-binding protein (ChREBP); cardiovascular disease (CVD); carnitine palmitoyl transferase-1 (CPT-1); Cranberries (CrBs); cranberries juice (CrB-JSB); CrB extracts (CrBE); CrB extracts (CrBEs); CrB juice (CrBJ); C-reactive protein (CRP); Diabetes mellitus (DM); diabetic retinopathy (DN); diacylglycerol acyltransferases 2 (DGAT2); fasting blood sugars (FBS); fatty acid synthase (FAS); Food and Agriculture Organization of the United Nations (FAO); forkhead box O1 (FOXO1); free fatty acid (FFA); gamma-glutamyltransferase (γ-GT); gastrointestinal-digested BB slurry (GIDBB); GB polysaccharides (GBPS); glucagon-like peptide-1 (GLP-1); glucose tolerance test (GTT); glucose transporter (GLUT4); glucose transporter 2 (GLUT-2); glucose-6-phosphatase, (G6Pase); glucose-stimulated insulin secretion (GSIS); glutathione (GSH); glycerol-3-phosphate acyltransferase (GPAT); glycogen synthase (GYS1); glycogen synthase 2 (GSY2); Goji berry (GB); high-carbohydrate diets, (HCD); high-fat diets, (HFD); high-sensitivity CRP (hs-CRP); Human aortic endothelial cells, (HAECs); human serum albumin (HSA); inducible nitric oxide synthase (iNOS); insulin receptor substrate-1/2 (IRS-1/IRS-2); insulin resistance, (IR); intercellular adhesion molecule-1 (ICAM-1); intestinal mucosal barrier dysfunction, (IMBD); lactate dehydrogenase (LDH); Lingonberry (LB); lipopolysaccharides (LPS); Low-calorie dried cranberry, (LCDC); low-density lipoprotein cholesterol (LDL-C); lysophosphatidylcholine (LPC); lyso-phosphatidylethanolamines, (LPE); malondialdehyde (MDA); manganese superoxide dismutase, (Mn-SOD); Maqui berries (MB); microbial-fermented blackberry metabolites (GMBB); microsomal TG transfer protein (MTP); mitochondrial transcription factor A (TFAM); monocyte chemo-attractant protein-1 (MCP-1); Mulberries (MBs); Na-glucose co-transporter 1 (SGLT-1); nitric oxide (NO); nitric oxides (NOs); nod-like receptor pyrin containing 3 (NLRP3); non-alcoholic fatty liver disease (NAFLD); paraoxonase-1 (PON-1); peroxisome proliferator response element (PPRE); peroxisome proliferator-activated receptors γ (PPAR-γ); phosphatidylcholines (PC); polyunsaturated fatty acid (PUFA); PPAR-γ coactivator 1α (PGC-1α); proinflammatory nuclear factor (NF)-κB; Raspberries (RBs); RB extracts (RBE); reactive oxygen species (ROS); short-chain fatty acids, (SCFA); short-chain organic acids, (SCOA); soluble vascular cell adhesion molecule-1 (sVCAM-1); sphingomyelins (SM); sterol regulatory element-binding protein 1c (SREBP-1c); streptozotocin (STZ); Toll-like receptors, (TLR); total cholesterol (TC); total glyceraldehyde (TG); Trolox equivalent antioxidant capacity (TEAC); Type 1 diabetes mellitus, (T1DM); type 2 diabetes mellitus, (T2DM); unsweetened dried CrBs (USCB); white bread (WB); World Health Organization, (WHO).
